# Gut microbiota and transcriptome dynamics in every-other-day fasting are associated with neuroprotection in rats with spinal cord injury

**DOI:** 10.3389/fmicb.2023.1206909

**Published:** 2023-07-28

**Authors:** Junyu Wang, Xiaohua Zhao, Ruihan Zhou, Meiyu Wang, Wu Xiang, Zilong You, Min Li, Ruiling Tang, Jingqi Zheng, Jiayu Li, Li Zhu, Jiaxin Gao, Huaqiang Li, Rizhao Pang, Anren Zhang

**Affiliations:** ^1^State Key Laboratory of Biotherapy, West China Hospital, Sichuan University, Chengdu, China; ^2^Department of Rehabilitation Medicine, General Hospital of Western Theater Command, Chengdu, China; ^3^Department of Rehabilitation Medicine, The People’s Hospital of Tongliang District, Chongqing, China; ^4^Rehabilitation and Wellness Care Centre, Tian Fu College of Swufe, Chengdu, China; ^5^Department of Biochemistry and Biophysics, School of Basic Medical Sciences, Peking University, Beijing, China; ^6^Department of Rehabilitation Medicine, Shanghai Fourth People’s Hospital Affiliated to Tongji University School of Medicine, Shanghai, China; ^7^School of Health Preservation and Rehabilitation, Chengdu University of Traditional Chinese Medicine, Chengdu, China

**Keywords:** every-other-day fasting, spinal cord injury, intermittent fasting, gut microbiota, transcriptome, neuroprotection

## Abstract

**Introduction:**

Every-other-day fasting (EODF) is a classical intermittent fasting (IF) mode with neuroprotective effects that promotes motor function recovery after spinal cord injury (SCI) in rats. However, its dynamic effects on the gut microbiota and spinal cord transcriptome remain unknown.

**Methods:**

In this study, 16S rRNA sequencing and RNA-seq analysis were used to investigate the effects of ad libitum (AL) and EODF dietary modes on the structural characteristics of rat gut microbiota in rats and the spinal cord transcriptome at various time points after SCI induction.

**Results:**

Our results showed that both dietary modes affected the bacterial community composition in SCI rats, with EODF treatment inducing and suppressing dynamic changes in the abundances of potentially anti-inflammatory and pro-inflammatory bacteria. Furthermore, the differentially expressed genes (DEGs) enriched after EODF intervention in SCI rats were associated with various biological events, including immune inflammatory response, cell differentiation, protein modification, neural growth, and apoptosis. In particular, significant spatiotemporal differences were apparent in the DEGs associated with neuroprotection between the EODF and AL interventions. These DGEs were mainly focused on days 1, 3, and 7 after SCI. The relative abundance of certain genera was significantly correlated with DEGs associated with neuroprotective effects in the EODF-SCI group.

**Discussion:**

Our results showed that EODF treatment may exert neuroprotective effects by modulating the transcriptome expression profile following SCI in rats. Furthermore, gut microbiota may be partially involved in mediating these effects.

## 1. Introduction

Spinal cord injury (SCI) is a highly disabling and traumatic condition without effective treatment options. In recent years, intermittent fasting (IF) has attracted increasing attention because it mediates the energy supply and avoids gastrointestinal damage caused by prolonged fasting (Hatting et al., 2017). Every-other-day fasting (EODF), a classic mode of IF, is a 24-h alternating fasting and feeding dietary pattern shown to exert neuroprotective effects; in studies of neurological diseases, EODF was found to reduce neuro-inflammation secondary to stroke and craniocerebral injury in experimental animals, thus exerting a neuroprotective effect and improving neurological deficits (Varady and Hellerstein, 2007; Davis et al., 2008; Manzanero et al., 2014). EODF-treated rat models of SCI recovered varying levels of motor and sensory function (Plunet et al., 2008, 2010; Jeong et al., 2011). Another study noted that in the SCI rat model, the first 24 h is a very acute phase that may involve changes in most immediate early stress genes; days 3 and 7 represent the peak of delayed apoptosis of neuronal cells; days 10–14 are deemed the subacute phase when tissue inflammation levels have subsided; and the subsequent period is considered the recovery period (Chamankhah et al., 2013). A comparison of two studies that screened for differential genes before and after SCI modeling revealed that the differentially expressed genes (DEGs) caused by SCI were mainly related to stress and immune response, with stress including an inflammatory response and immune response including innate and adaptive immune response; these studies showed that the immune and inflammatory responses continued to spread from the acute phase to the chronic phase, with an intrinsic immune response dominating in the acute phase and an adaptive immune response dominating in the subacute and chronic phases (Chamankhah et al., 2013; Shi et al., 2017). These results suggest that the inflammatory and immune responses are the predominant biological events following SCI. We previously found that EODF exerted significant neuroprotective effects in a rat model of SCI, promoting the recovery of motor function, reducing the inflammatory response in the plane of injury, and inhibiting apoptosis and necrosis (Sun et al., 2018; Li J. J. et al., 2022). However, at the genetic level, the mechanisms by which EODF regulates gene expression levels in distal spinal cord tissue have not been clarified. Furthermore, the biological mechanisms by which EODF exerts its neuroprotective effects in a rat model of SCI treatment are not fully understood.

SCI can disrupt the balance of the gut microbiota (Gungor et al., 2016; Kigerl et al., 2016). The diversity and structural composition of gut microbiota were reduced in SCI patients, suggesting an association between the neurogenic rectum and the gut microbiota (Zhang et al., 2018). SCI has been shown to promote intestinal dysfunction and mucosal permeability, leading to translocation of the gut microbiota (Kigerl et al., 2016). We previously described disturbances in the gut microbiota of patients with SCI and found a correlation in the abundance of certain microbial genera and lymphocyte subpopulations (Pang et al., 2022). Gut microbiota plays key roles in immune, nutritional, and metabolic functions (Ejtahed et al., 2017). Some diet therapies can restore or dramatically alleviate dysfunctions in gut microbiota and are considered a promising approach to preventing and treating global health problems. Increasing evidence indicates that IF has a variety of health benefits for individuals that are healthy (Ali et al., 2021; Su et al., 2021, 2022), obese (Li et al., 2017; Deng et al., 2020; Liu et al., 2021a), or diabetic (Liu et al., 2020), as well as those with cardiovascular disease (Guo et al., 2021; Prisco et al., 2021) hypertension (Maifeld et al., 2021; Shi H. et al., 2021), and neurological disorders (Cignarella et al., 2018; Serger et al., 2022). The beneficial effects of IF are considered to be exerted through the restoration of gut microbiota and metabolite production; however, to the best of our knowledge, the characteristics of gut microbiota in SCI rats treated with EODF have not been reported to date.

**Figure 1:**
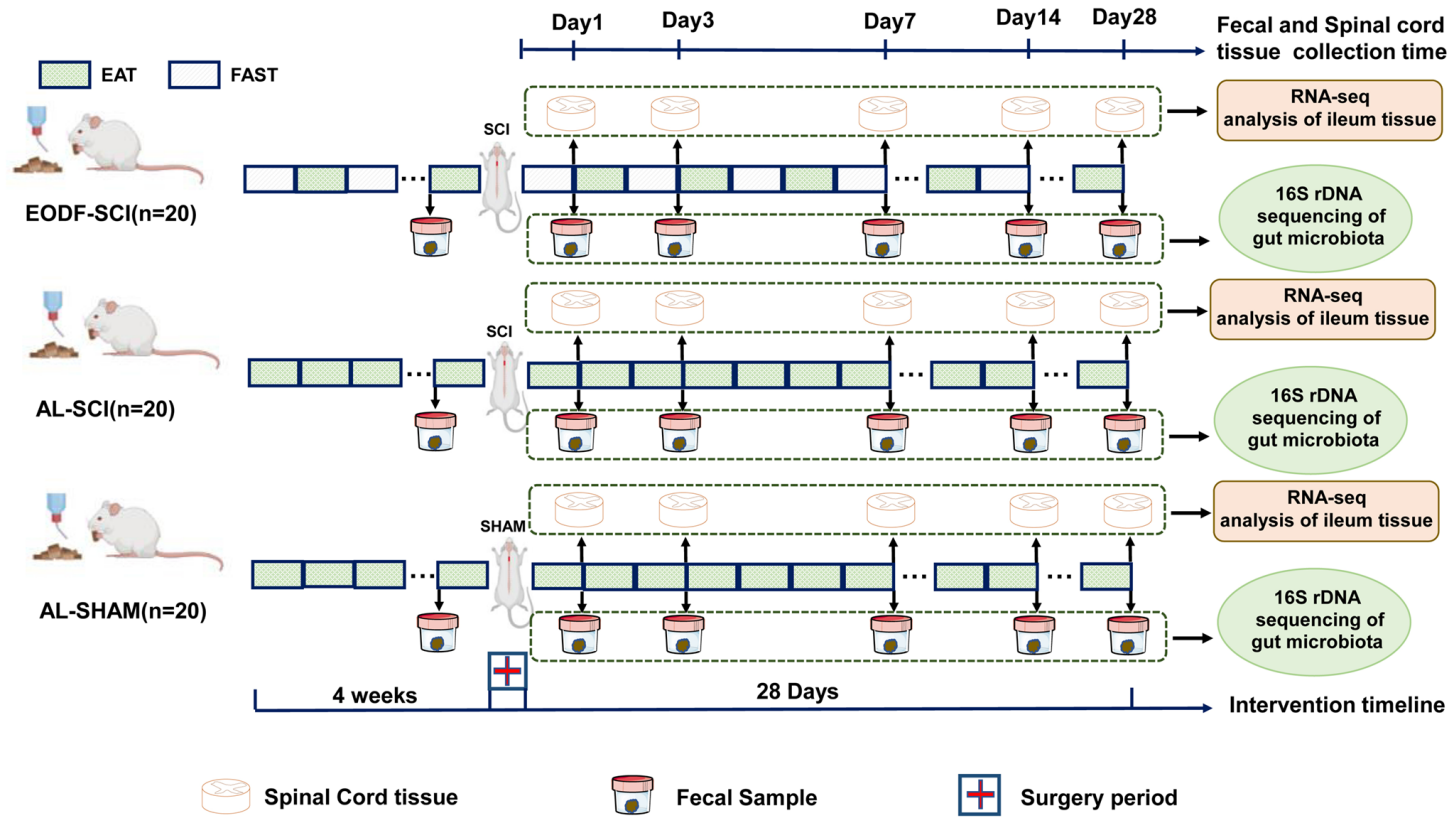
Experimental grouping and design.

To further understand the effects of EODF on the gut microbiota and spine cord tissue transcriptome in SCI rats during the acute, subacute, and recovery phases, we designed the following experiments (Figure 1). We assessed the dynamic changes of gut microbiota in an *ad libitum* diet (AL)-SCI and EODF-SCI group based on the 16S rRNA high-throughput sequencing of fecal samples collected 1 day before and 1, 3, 7, 14, and 28 days after modeling. Furthermore, we assessed the dynamic changes of DEGs in the *ad libitum* diet (AL)-SCI and EODF-SCI groups based on transcriptomic data collected from spinal cord tissues after 1, 3, 7, 14, and 28 days and biological annotation of DEGs through Gene Ontology (GO) and Kyoto Encyclopedia of Genes and Genomes (KEGG) databases to explore the effects of EODF on gene transcription in post-injury spinal cord tissues, particularly on neuroprotection-related DEGs and pathways after SCI. Finally, we used the Spearman^’^s correlation coefficient to identify the gut microbiota associated with neuroprotection-related DEGs. Accordingly, we aimed to describe the characteristics of gut microbiota and DEGs associated with each diet mode and identify the critical bacteria, key DEGs, and main biological events associated with neuroprotection.

## 2. Materials and methods

### 2.1. Animal grouping

Healthy male Sprague–Dawley rats (2 months old,130 ± 10 g) were purchased from Chengdu Dashuo Experimental Animal Co., (Chengdu, China). These rats were housed in standard plastic cages (4 animals per cage) in a quiet, clean animal room under temperature control (22 ± 2°C) and a 12-h light–dark cycle (lights were turned on at 8:00 am and turned off at 8:00 pm). To determine the effects of EODF on the gut microbiota of healthy rats, we randomly divided 12 rats into two groups: an experimental group (EODF-Healthy) and a control group (AL-Healthy). To study the effect of EODF on the gut microbiota and spinal cord tissue transcriptome data of SCI rats, we randomly divided 40 successfully modeled SCI rats into the AL-SCI and EODF-SCI groups, in addition, 20 rats were sham-operated as a control group. To study the effect of EODF on the recovery of motor function in SCI rats, we randomly divided 32 successfully modeled SCI rats into AL-SCI and EODF-SCI groups for behavioral analysis, while 16 rats performed sham surgery as a control group.

### 2.2. SCI and SHAM modeling

An intravenous dose of sodium pentobarbital (45 mg/kg) was used to anesthetize the rats, after which hair was plucked from the back of the neck and the cleared region was cleaned with iodophor. Beginning at T2 of the thoracic spine, a 3–4 cm incision was made along the head and neck. To properly uncover the C4–C6 vertebral plates of the cervical spine, the surrounding fascia, muscles, and ligaments of the neck had to be dissected apart one by one. It was possible to see the spinal cord, the central vein of the dorsal median sulcus, and both C6 nerve roots after opening the C5 vertebral plate using biting forceps. Special care was taken not to break the dura mater during surgery, which is Located perpendicular to the long axis of the spinal column and above the C6 nerve root. After applying a 70 g closure force with a temporary aneurysm clamp (Yasargil Titanium Mini-Clips, Germany) on the spinal cord epidurally for 30 s, the clamp was withdrawn to a depth ranging from the periphery of the spinal cord to the dorsal median sulcus (C5 half of the spinal cord). For consistency, the same person clamped the spinal cord in every animal used in the experiments. After the incisions had been cleaned and disinfected, the muscles and skin were then sutured together, one layer at a time. The recuperating rats were housed in an incubator until they had totally awoken and were active. Dehydration was avoided by injecting intraperitoneal normal saline (5 mL/animal) for 2 days following surgery. When the injured rat displayed paralysis in the ipsilateral front paw, when the damaged ipsilateral forelimb had difficulties straightening forward and downward when the tail was elevated in the air, or when it fell backward toward the afflicted side when walking, the model was deemed effective (Supplementary Figure S1). In the SHAM-operated group, the steps were the same as those in the SCI group, except that no spinal cord tissue was damaged.

### 2.3. Behavioral analysis

We referred to a previous method to assess the behavior of SCI rats, whereby the grooming, horizontal ladder, and cylinder rearing tests were used to evaluate the motor function of the affected forelimb in rats (Streijger et al., 2014). Two weeks before modeling, all rats were trained to familiarize them with the behavioral evaluation. Three groups of rats (*n* = 16/group) were tested using these three tests 1 day before surgery, 1 day after surgery, and 1, 2, 4, 6, 8, 10, and 12 weeks after surgery.

### 2.4. Fecal sample and spine cord tissues collection

Throughout the study, the AL-Healthy group had free access to food and water; whereas, the EODF-Healthy group was fed and fasted every other 24 h. Fecal samples were collected on day 28 after initiating the dietary intervention. Rats in the AL-SCI and AL-SHAM groups had unrestricted access to food and water; whereas, those in the EODF-SCI group began the EODF diet 4 weeks prior to surgery and were fed and fasted every 24 h. In the AL-SCI, AL-SHAM, and EODF-SCI groups, after 1, 3, 7, 14, and 28 days post-operation, rats were euthanized to collect spinal cord tissue. Four rats were randomly selected at each time point. After exposing the spinal cord tissue, approximately 0.5 cm of fresh spinal cord tissue (1 cm in total) was collected cephalad and caudal to the C5 injury area to meet the needs of the test. The surface blood cells were washed off with Phosphate Buffered Saline, placed in a lyophilization tube, labeled, and placed in liquid nitrogen. The entire collection process was carried out in a cold environment. The room temperature was kept below 25°C, and the time between spinal cord tissue release and placement in liquid nitrogen was kept to a minimum of 10 min. After collection, all specimens were quickly frozen in a − 80°C refrigerator and stored. Fecal samples were collected 1 days before and 1, 3, 7, 14, and 28 days after modeling, and 0.5–1 g of the central portion of each fecal sample was placed in a germ-free fecal collection tube. To maintain a somewhat anaerobic environment, the tube cap was quickly closed after sample collection. Fecal samples were kept at −80°C until further examination. The rats were in an unfed state when the spinal cord tissue and intestinal feces were collected.

### 2.5. 16S rRNA sequencing

The Power Fecal DNA Extraction Kit (Qiagen, Hilden, Germany) was used to extract DNA from fecal samples. The 16S rRNA V4 region of each sample was amplified. The relative bands were recovered for 16S rRNA sequencing using a gel recovery kit after electrophoresis of mixed PCR results from the same sample (Qiagen, Hilden, Germany). The primers used were 515F (5′-GTGC CAGCMGCCGC GGTAA-3′) and 806R (5′-GGACTACHVGGGTWT CTAAT-3′; Caporaso et al., 2011; Liu et al., 2017). The sequencing was carried out utilizing the Illumina HiSeq sequencing technology (Illumina, San Diego, CA, United States).

### 2.6. RNA-seq analysis of spinal cord tissues

Using the protocol provided by the manufacturer of the mirVana™ miRNA ISOlation Kit (Ambion-1561), total RNA was isolated from spinal cord tissue (*n* = 4 per group). The 2100 Bioanalyzer (Agilent Technologies, United States) was used to evaluate total RNA quality, and a NanoDrop ND-2000 (Thermo Scientific, United States) was used for quantification. Libraries for RNA sequencing were made using TruSeq™ RNA Sample Preparation Kits (Illumina, United States) and were quality-checked again with a 2100 Bioanalyzer (Agilent Technologies, United States) before processing on an Illumina HiSeq™ 2500 (Illumina, United States) for sequencing.

### 2.7. Bioinformatics analysis

#### 2.7.1. Gut microbiome

For the operational taxonomic unit (OTU) analysis, all sequences were classified using UPARSE software version 7.1 (Edgar, 2013). Those with >97% similarity were clustered into one OTU, and then the OTUs were filtered using SSU115.^1^ We assessed α diversity according to species richness (ACE and Chao1) and diversity indices (Shannon and Simpson). Principal coordinate analysis (PCoA) was used to examine beta-diversity using the unweighted UniFrac method. The typical OTU sequences were classified using a Bayesian technique for nucleic acid database classification for subsequent taxonomic analysis.

#### 2.7.2. Spinal cord tissue transcriptome

Low-quality bases were removed, and high-quality clean reads were obtained by using the NGS QC Toolkit program for quality control of raw reads. Genomic alignment was performed using the Hisat2 (hierarchical indexing for spliced alignment of transcripts) program (Kim et al., 2015). Transcript expression was calculated using FPKM (fragments per kb per million reads; Roberts et al., 2011) and assessed using Cufflinks software to quantify gene abundance and identify the genes for subsequent analysis. The R language DEseq2 (Love et al., 2014) was used to normalize the number of counts of genes in each sample. After multiplexing the false discovery rate (FDR), *p* < 0.05 and |log2(foldchange)| > 0.58 were DEGs. Functional enrichment of DEGs was analyzed using the Gene Ontology (GO)^2^ and Kyoto Encyclopedia of Genes and Genomes (KEGG)^3^ databases.

### 2.8. Statistical analysis

SPSS 19.0 was used for all statistical analyses. We used various methods to test whether the data were normally distributed, including the Shapiro–Wilk test, skewness–kurtosis test, and a graphical method (P–P plot and Q-Q plot), and made our determination based on whether the results of multiple methods were in agreement. If two or more methods showed that the data obeyed the normal-terrestrial distribution, the data were considered to be normally distributed; otherwise, they were considered to be non-normally distributed. Data with a normal distribution and uniform variance were analyzed by one-way ANOVA (LSD method for two-way comparison) or t-test for two independent samples; The Kruskal–Wallis rank–sum test (Dunn’s method for two-by-two comparisons and Bonferroni correction for test results) or the Mann–Whitney U test were used if they did not obey a normal distribution or if the variance was not uniform. All graphs were generated using Graphpad Prism9.0 or Chiplot.^4^ *p* < 0.05 was chosen as the level of statistical significance.

## 3. Results

### 3.1. 16S rRNA gene sequencing results

For all fecal samples collected from the EODF-Healthy, AL-Healthy, AL-SCI, AL-SHAM, and EODF-SCI groups, 2505001raw and 2,467,372 clean reads with an average length of 282 bp were produced by 16S rRNA gene sequencing. Using dilution curves (“abundance curves”), we verified that the amount of sequencing data for each group was sufficient to reflect the species diversity (Supplementary Figure S2).The curves leveled off and reached a plateau for all samples. We concluded that the sequencing depth would cover all species in the samples, and we subsequently clustered the spliced tags into OTUs.

### 3.2. EODF treatment improves locomotor recovery in SCI (C5 half of the spinal cord) rats

We investigated the effects of EODF on locomotor recovery in rats with SCI (C5 half of the spinal cord). Locomotor recovery was observed during the 12 weeks post-injury in the SCI groups. EODF treatment further significantly increased locomotor function starting from 4 weeks (grooming test), 8 weeks (horizontal ladder test), and 10 weeks (cylinder rearing test) after injury compared to that of the SCI groups. The improvement in grooming test scores, ipsilateral forelimb errors, and contact with ipsilateral forelimb continued until the end of the experiment (Supplementary Figures S1B–D).

### 3.3. Analysis of the α and β diversity of gut microbiota

**Figure 2:**
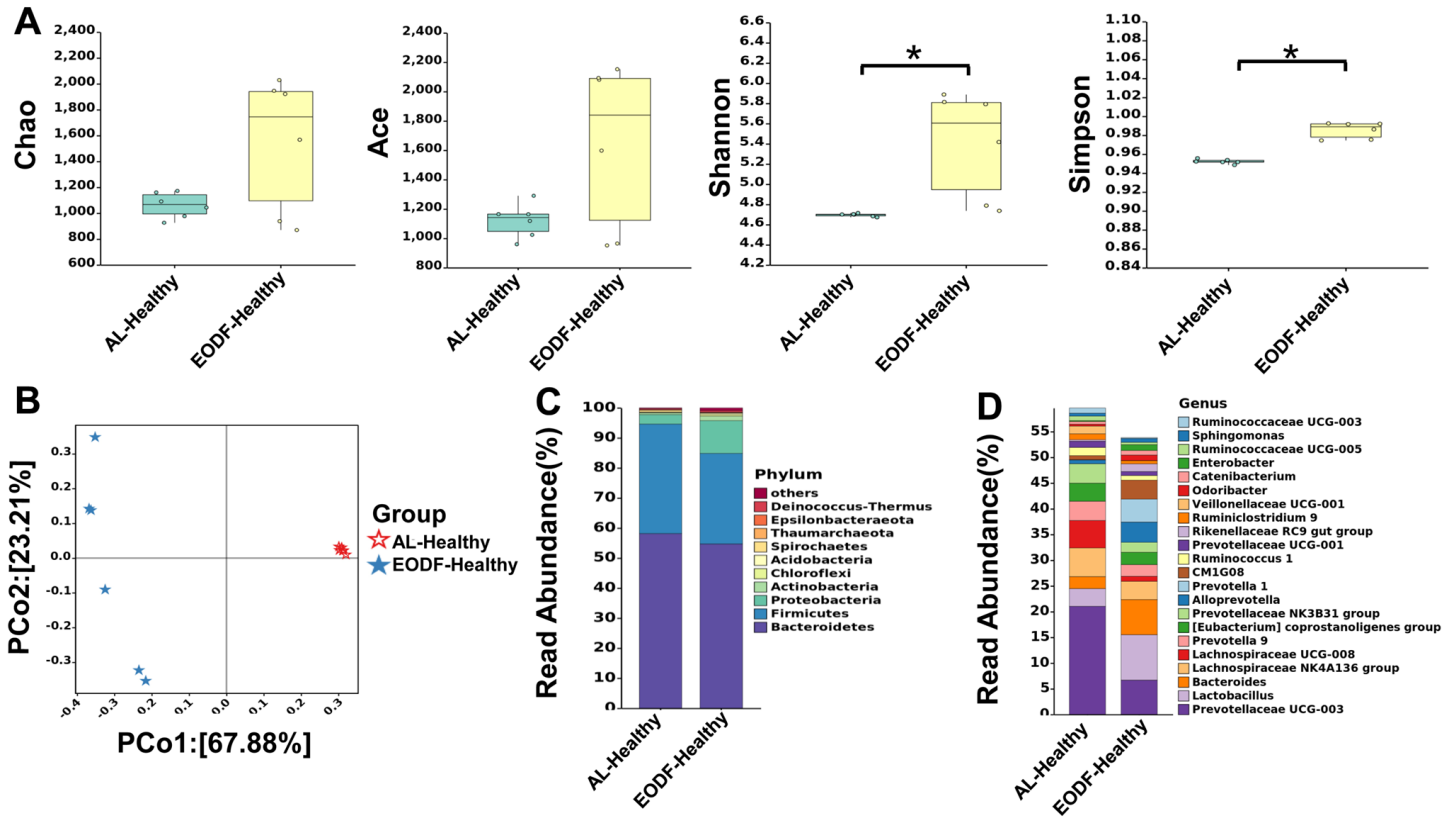
Effect of the *ad libitum* diet (AL) and every-other-day fasting (EODF) on gut microbiota diversity and structural composition in healthy rats. **(A)** Chao 1, ACE, Shannon, and Simpson indices assessing the difference in α-diversity between the two healthy groups (*n* = 6/group). Data were analyzed using the Mann–Whitney U test,*p < 0.05. **(B)** Principal coordinate analysis assessing the difference in β-diversity between the two healthy groups (*n* = 6). **(C)** Compositional distribution of gut microbiota phyla between the two healthy groups (*n* = 6). **(D)** Compositional distribution of gut microbiota genera between the two healthy groups (*n* = 6).

**Figure 3:**
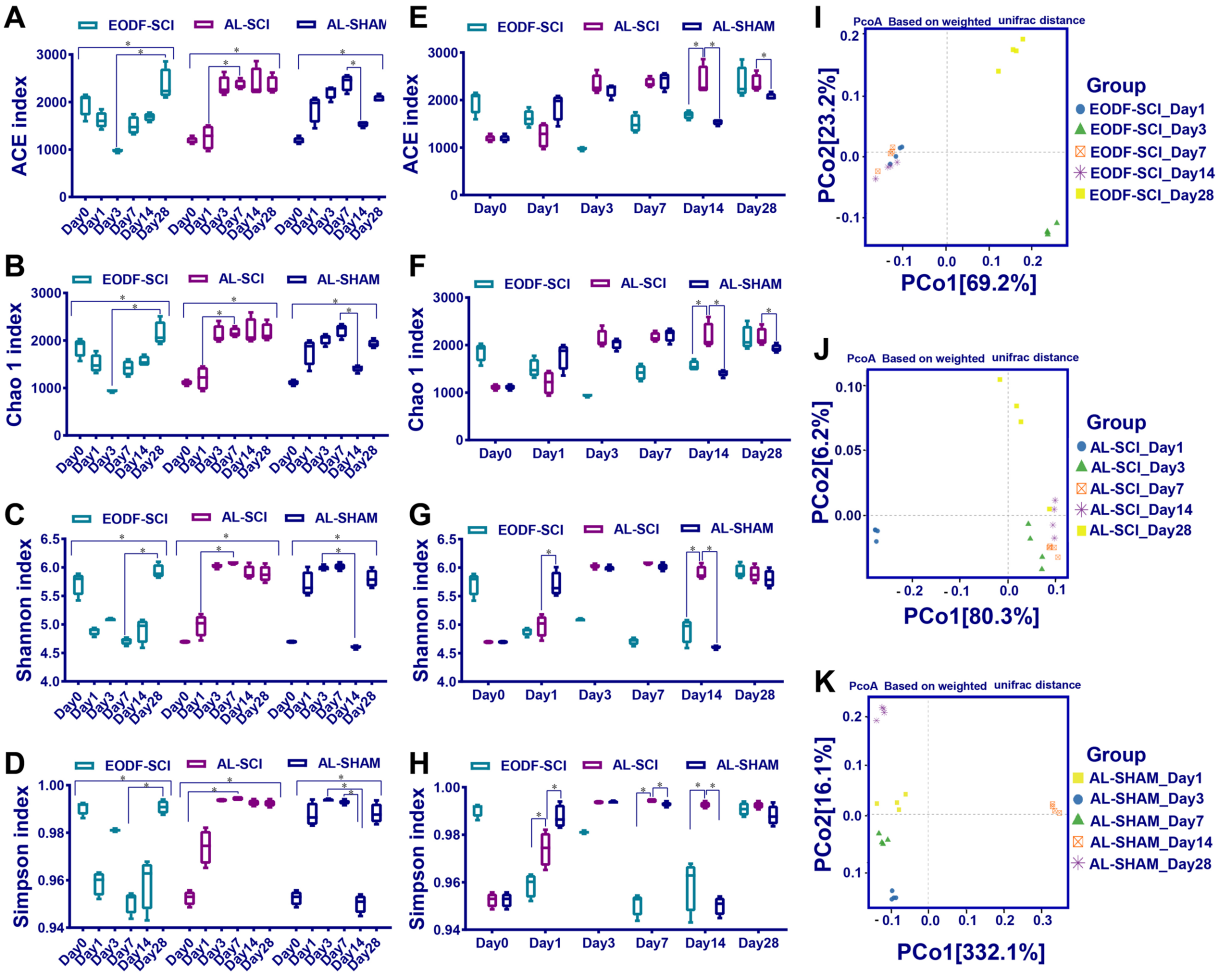
Changes in gut microbiota diversity over time. **(A–D)** Chao 1, ACE, Shannon, and Simpson indices assessing α-diversity over time between the EODF-SCI, AL-SCI, and AL-SHAM groups. Data were analyzed using the Kruskal–Wallis test with Dunn *post-hoc* tests. **(E–H)** Chao 1, ACE, Shannon, and Simpson indices assessing α-diversity at the same time points between the EODF-SCI, AL-SCI, and AL-SHAM groups. Data were analyzed using the Mann–Whitney U test. *N* = 4, *indicates that indices were significantly higher (*p <* 0.05). **(I–K)** Principal coordinate analysis assessing β-diversity over time in each group (*n* = 4).

First, we compared the effects of EODF and AL on the α diversity of the gut microbiota in healthy rats (Figure 2A). The diversity of gut microbiota in the EODF-Healthy group was significantly higher than that of the AL-Healthy group. However, there was no significant difference in gut microbiota abundance between the two groups. According to the PCoA (Figure 2B), the OTUs of the two groups showed dispersion and aggregation, indicating that β diversity varied between the two groups. We compared the patterns in gut microbiota α diversity between the EODF-SCI, AL-SCI, and AL-SHAM groups on days 0, 1, 3, 7, 14, and 28. As shown in Figures 3A–D, there were significant differences in the Ace, Chao 1, Shannon, and Simpson indices between pre- and post-modeling samples in these three groups. In the EODF-SCI group, ACE and Chao 1 indices decreased exponentially on days 1 and 3 after SCI injury, and recovered gradually until day 28. Shannon and Simpson indices decreased on day 1 after SCI injury, increased gradually until day 3, decreased on day 7, and recovered gradually until day 28. In the AL-SCI group, ACE, Chao 1, Shannon, and Simpson indices increased exponentially on day 3 after SCI injury, and remained stable until day 28. In the AL-SHAM group, ACE, Chao1, Shannon, and Simpson indices increased gradually on day 1 after SCI injury until day 7, decreased exponentially on day 14, and increasing on day 28. These results indicated that the species richness and diversity of the gut microbiota in the three groups had different characteristics.

The α diversity of the bacterial community at each time point was compared between the EODF-SCI, AL-SCI, and AL-SHAM groups. As shown in Figures 3E–H, on day 14 after SCI, the Chao 1 and ACE indices were significantly lower in the EODF-SCI group compared to those in the AL-SCI group, suggesting that EODF intervention reduced the richness of the gut microbiota in SCI rats at that time point. On days 1, 7, and 14 after SCI, the Simpson index was significantly lower in the EODF-SCI group compared to that in the AL-SCI group, suggesting that the EODF intervention reduced the diversity of the gut microbiota of SCI rats in these time points. Furthermore, we observed a significant decrease in the Shannon index in the EODF-SCI group on day 14.

According to the PCoA, days 1, 7, and 14 were clustered together in the EODF-SCI group; days 3, 7, and 14 were clustered in the AL-SCI group; and days 1, 3, and 7 were clustered in the AL-SHAM group (Figures 3I–K). These results indicated that gut microbiota diversity changed over time, and that different dietary interventions had different effects on the β diversity of gut microbiota in rats with SCI.

### 3.4. Phylum- and genus-level composition of gut microbiota

We explored the effects of the two dietary interventions on the levels of gut microbiota phyla in healthy rats and found no difference between rats in the EODF-Healthy and AL-Healthy rats (Supplementary Figure S3A). *Bacteroidetes* and *Firmicutes* were the dominant phyla in these groupis (Figure 2C). The relative abundances of *Bacteroidetes* and *Firmicutes* in the AL-Healthy group were 58.17 and 36.42%, and those in the EODF-Healthy group were 54.72 and 30.08%, respectively. In the EODF group, the abundance of *Firmicutes* was significantly reduced (30.08%).

**Figure 4:**
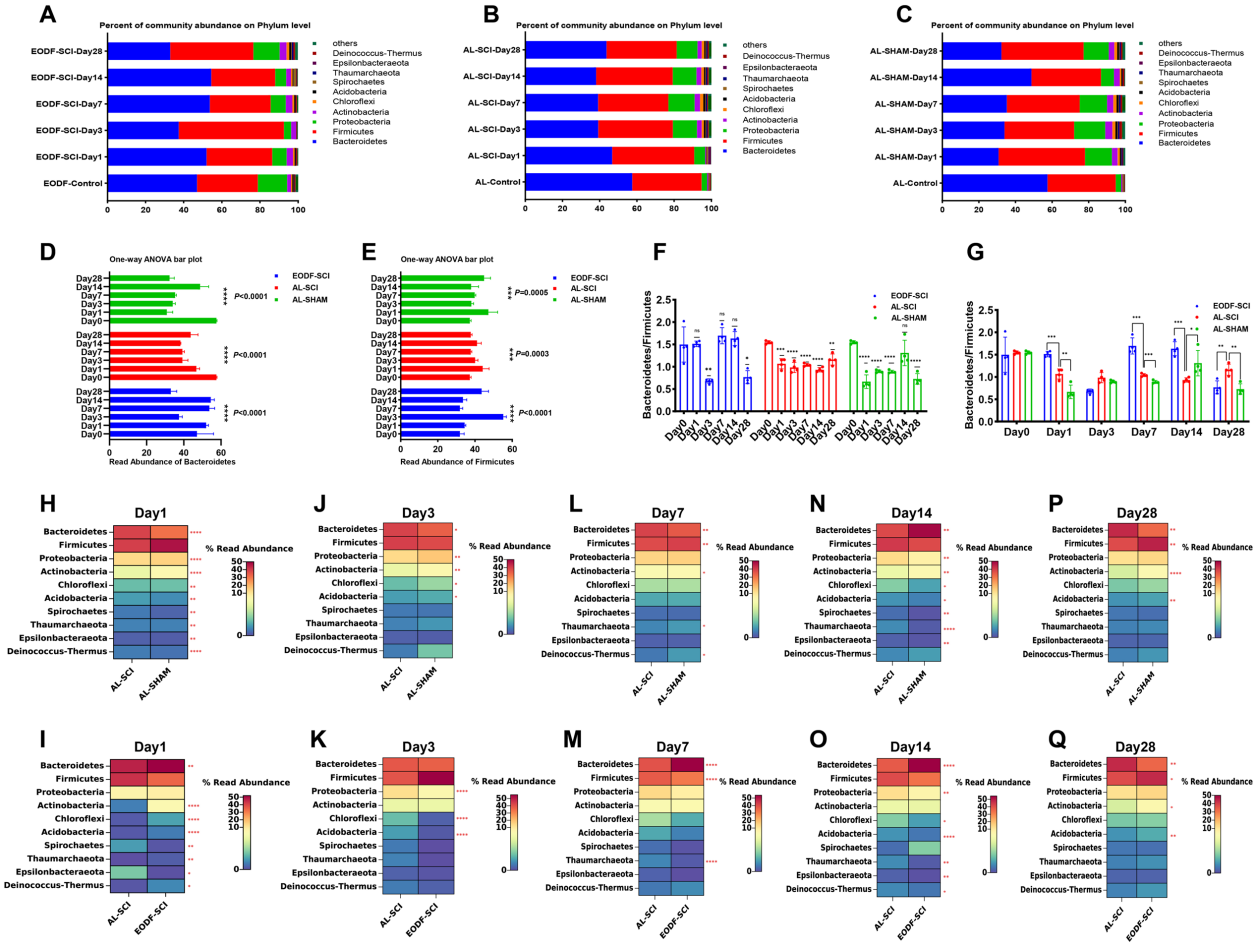
Gut microbiota composition at the phylum level. Comparison of the bacterial community composition at the phylum-level and changes over time in the: **(A)** EODF-SCI, **(B)** AL-SCI, **(C)** AL- SHAM groups. Microbial abundance was calculated as a percentage of the total bacterial taxon within each sample. The bar chart shows the average value for each group. Further analysis of the relative abundances of *Bacteroides* and *Firmicutes* over time between the EODF-SCI, AL-SCI, and AL- SHAM group; significance test of the relative abundance of: **(D)** *Bacteroides* and **(E)** *Firmicutes* using one-way ANOVA. **(F)** Comparison of the *Bacteroidetes*/*Firmicutes* ratio at pre-modeling (day 0) and post-modeling (days 1, 3, 7, 14, and 28) using a Student’s *t*-test. **(G)** Comparison of the *Bacteroidetes*/*Firmicutes* ratios at the same time points between the EODF-SCI, AL-SCI, and AL-SHAM groups using a Student’s *t* test. **(H–P)** Heatmap showing differential bacteria at the phylum level at the same time points between the AL-SCI and AL-SHAM groups using a Student’s *t* test. **(I–Q)** Heatmap showing differential bacteria at the phylum level at the same time points between the EODF-SCI and AL-SCI groups using a Student’s *t* test. *n* = 4, *p < 0.05, **p < 0.01, ***p < 0.001, ****p < 0.0001.

To understand the effects of the two dietary interventions on changes in the structure of the gut microbiota in SCI rats, we analyzed the distribution of the gut microbiota at six time points. At the phylum level, the most abundant phyla in the EODF-SCI, AL-SCI, and AL-SHAM groups were *Firmicutes* and *Bacteroidetes* (Figures 4A–C). We then used a one-way ANOVA to assess the effect of the two interventions on changes in *Firmicutes* and *Bacteroidetes* abundance at six time points (Figures 4D,E). In the EODF group, the *Bacteroidetes* abundance significantly decreased on day 3 after SCI, then increased on days 7 and 14 post-injury, and finally decreased on day 28; whereas, the *Firmicutes* abundance significantly increased on day 3 after SCI, then decreased on days 7 and 14 post-injury, and finally increased on day 28. In the AL-SCI group, the *Bacteroidetes* abundance gradually decreased on day 1 after SCI, then remained stable on days 3, 7, and 14 after injury, and finally increased slightly on day 28; whereas, *Firmicutes* abundance slowly increased on day 1 after SCI, then remained stable on days 3, 7, 14, and 28 after injury. In the AL-SHAM group, the *Bacteroidetes* abundance decreased significantly on day 1 after SCI, remained stable on days 3 and 7 after injury, increased significantly on day 14, and then decreased on day 28; whereas, the *Firmicutes* abundance increased slightly on day 1 after SCI, then remained stable on days 3, 7, and 14 after injury, and finally increased slightly on day 28.

As represented in Figure 4F, we analyzed the changes in the *Bacteroidetes*/*Firmicutes* ratio. In the AL-SCI group, we found that this ratio decreased significantly on day 1 until day 28 after SCI. This findings suggested that SCI led to time-dependent gut microbial dysbiosis. In the AL-SHAM group, this ratio decreased significantly on days 1, 3, 7, and 28; whereas, in the EODF-SCI group, it only decreased significantly on days 3 and 28 after SCI. Interestingly, we found a significant increase in the *Bacteroidetes*/*Firmicutes* ratio in the EODF-SCI group on days 1, 7, and 14 after injury and a significant decrease on day 28 compared to that in the AL-SCI group (Figure 4G). We further analyzed the differences in the phylum level bacterial populations at the same time point in the EODF-SCI and AL-SCI groups. As shown in Figures 4H–Q, on day 1 after injury, the abundances of *Bacteroidetes*, *Actinobacteria*, *Chloroflexi*, *Acidobacteria*, *Thaumarchaeota*, and *Deinococcus*-*Thermus* significantly increased, whereas those of *Spirochaetes* and *Epsilonbacteraeota* decreased, compared to those in the AL-SCI group. On day 3 after injury, the abundances of *Proteobacteria*, *Chloroflexi*, and *Acidobacteria* were significantly lower than those in the AL-SCI group. On day 7 after injury, the *Bacteroidetes* abundance was significantly increased, and the abundances of *Firmicutes* and *Thaumarchaeota* were significantly decreased compared to those in the AL-SCI group. On day 14 after injury, the *Bacteroidetes* abundance was significantly increased, and the abundances of *Proteobacteria*, *Chloroflexi*, *Acidobacteria*, *Thaumarchaeota*, *Epsilonbacteraeota*, and *Deinococcus-Thermus* were significantly decreased compared to those in the AL-SCI group. On day 28 after injury, the abundances of *Firmicutes*, *Actinobacteria*, and *Acidobacteria* increased significantly and that of *Bacteroidetes* decreased significantly compared to those in the AL-SCI group. These findings suggested that EODF appeared to dynamically regulate changes in the gut microbiota of SCI rats.

We compared the differences in gut microbiota between the EODF-Healthy and AL-Healthy groups at the genus level (Supplementary Figure S3B). Compared to the AL-Healthy group, the abundances of the genera *Lactobacillus, Bacteroides, Alloprevotella, Prevotella 1, Rikenel-laceae RC9, Odoribacter, and Catenibacterium* spp. were significantly increased in the EODF-Healthy group, whereas that of *Prevotellaceae UCG-003, Lachnospiraceae NK4A136, Lachnospiraceae UCG-008, Prevotella 9, [Eubacterium] coprostanoligenes, Ruminococcaceae UCG-008, and Ruminococcaceae UCG-003*(among others) was significantly reduced (Figure 2D; Supplementary Table S1).

**Figure 5:**
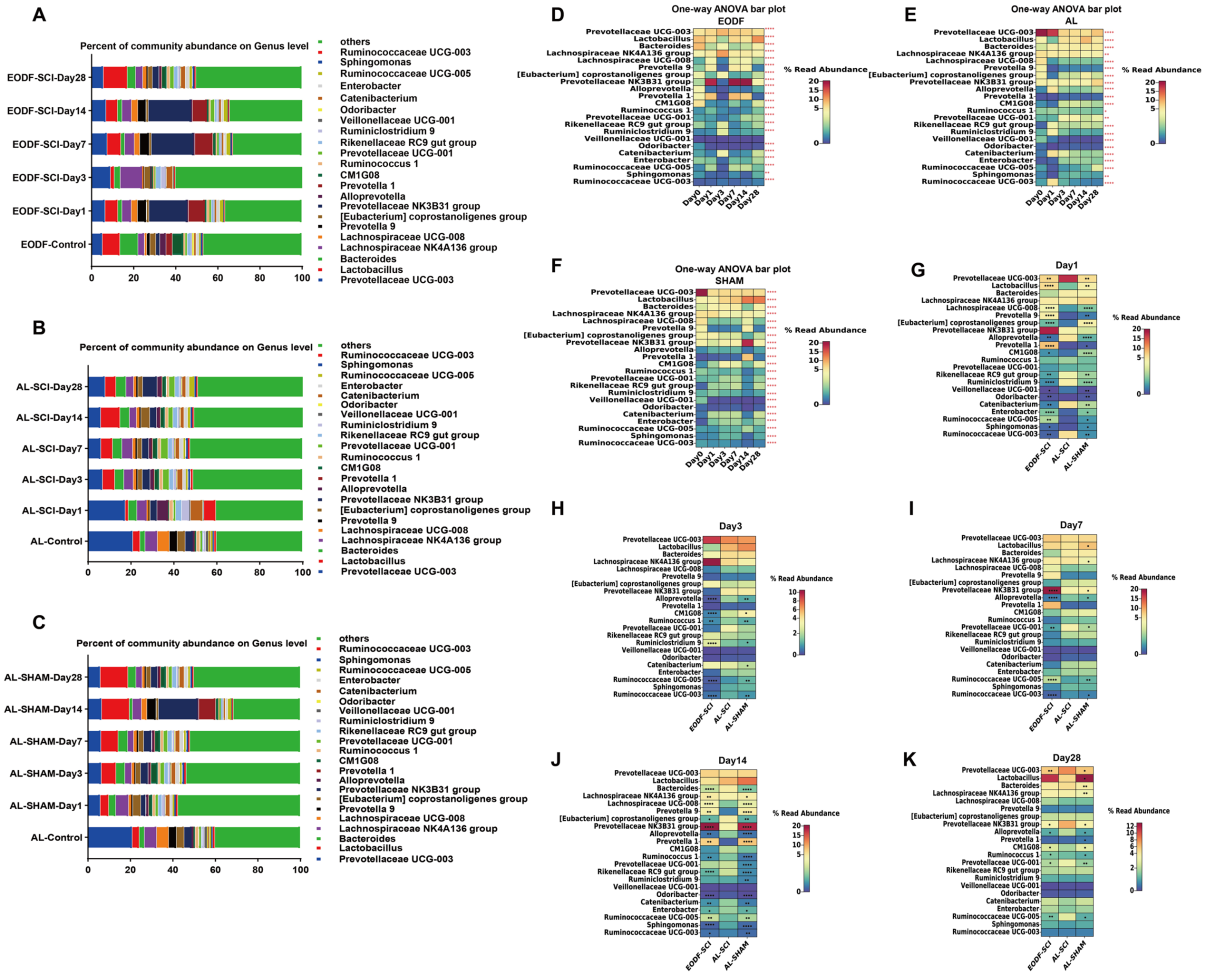
Gut microbiota composition at the genus level. Comparison of the bacterial community composition at the genus level and changes over time in the: **(A)** EODF-SCI; **(B)** AL-SCI; **(C)** AL- SHAM groups. Microbial abundance was calculated as a percentage of the total bacterial taxon within each sample. The bar chart shows the average value for each group. Further analysis the relative abundance of genus level over time between the three groups. Significance test of the relative abundances of: **(D)** EODF-SCI, **(E)** AL-SCI, and **(F)** AL-SHAM groups using one-way ANOVA. **(G–K)** Heatmap showing differential bacteria at the genus level at the same time points between three groups using a Student’s *t* test. Asterisks in the heat map squares indicate that the EODF-SCI and AL-SHAM groups were significantly different from AL-SCI group. *N* = 4, *p < 0.05, **p < 0.01, ****p < 0.0001.

To understand the effects of the two dietary interventions on structural changes in the gut microbiota of SCI rats, we analyzed the genus-level (top 22) gut microbiota distribution at six time points (Figures 5A–C). We then used a one-way ANOVA to assess the effect of the two interventions on changes in the genus-level (top 22) gut microbiota at six time points (Figures 5D–F). We found no significant difference in the *Veillonellaceae UCG-001* abundance in the EODF-SCI group; however, the abundances of all other bacteria differed significantly in all three groups. We further analyzed the differences in the genus level bacterial populations at the same time point in the EODF-SCI and AL-SCI groups. As shown in Figures 5G–K, on the first day after injury, the abundances of *Lactobacillus, Lachnospiraceae UCG-008, Prevotella9, [Eubacterium]coprostanoligenes group, Prevotella1, CM1G08, Odoribacter, Enterobacter, RuminococcaceaeUCG-005,* and *Sphingomonas* increased; whereas, the abundances of *Prevotellaceae UCG-003, Alloprevotella, Rikenellaceae RC9 gut group, Ruminiclostridium 9, Veillonellaceae UCG-001, Catenibacterium,* and *Ruminococcaceae UCG-003* decreased, compared to those in the AL-SCI group. On day 3 after injury, the abundance of *Ruminiclostridium 9* increased; whereas, the abundances of *Alloprevotella, CM1G08, Ruminococcus 1, Ruminococcaceae UCG-005,* and *Ruminococcaceae UCG-003* decreased compared to those in the AL-SCI group. On day 7 after injury, the abundances of *Prevotellaceae NK3B31* and *Ruminococcaceae UCG-005 group* increased, whereas those of *Alloprevotella, Prevotellaceae UCG-001,* and *Ruminococcaceae UCG-003* decreased, compared to those of the AL-SCI group. On day 14 after injury, the abundances of *Lachnospiraceae NK4A136 group, Lachnospiraceae UCG-008, Prevotella 9, Prevotella 1, Prevotellaceae NK3B31,* and *Ruminococcaceae UCG-005 group* increased, whereas those of *Bacteroides, [Eubacterium] coprostanoligenes group, Alloprevotella, Ruminococcus 1, Rikenellaceae RC9 gut group, Odoribacter, Catenibacterium, Enterobacter, Sphingomonas,* and *Ruminococcaceae UCG-003* decreased, compared to those of the AL-SCI group. On day 28 after injury, the abundances of *CM1G08* and *Ruminococcus 1* increased, whereas those of *Prevotellaceae UCG-003, Prevotellaceae NK3B31, Alloprevotella, Prevotellaceae UCG-001,* and *Ruminococcaceae UCG-005* decreased, compared to those of the AL-SCI group. These results provide further evidence that EODF intervention led to a dynamic gut microbiota profile in SCI rats that differed from that after AL intervention.

We analyzed the relative abundance and variations of potentially anti-inflammatory genera (including *Prevotella, Lactobacillus, and Lachnospiraceae*) and pro-inflammatory genera (*Bacteroides*), which showed dynamic changes in the AL and EODF groups. On day 1 after SCI, there were significant increases in the abundances of *Lactobacillus*, *Prevotella1*, *Prevotella9*, and *Lachnospiraceae UCG-008* in the EODF-SCI group compared to those in the AL-SCI group. On day 14 after SCI, there were significant increases in the abundances of *Prevotella1, Prevotella9*, and *Lachnospiraceae UCG-008* in the EODF-SCI group compared to those in the AL-SCI group, However, there was a significant decrease in the *Bacteroide* abundance. These results indicated that EODF treatment induced dynamic changes in the abundance of potentially anti-inflammatory and pro-inflammatory bacteria.

In summary, these results suggested that EODF regulated the relative abundance and variation of gut microbiota, leading to changes in the dynamics of bacteria that differed from those of the AL group.

### 3.5. Transcriptomic analysis of spinal cord tissue

**Figure 6:**
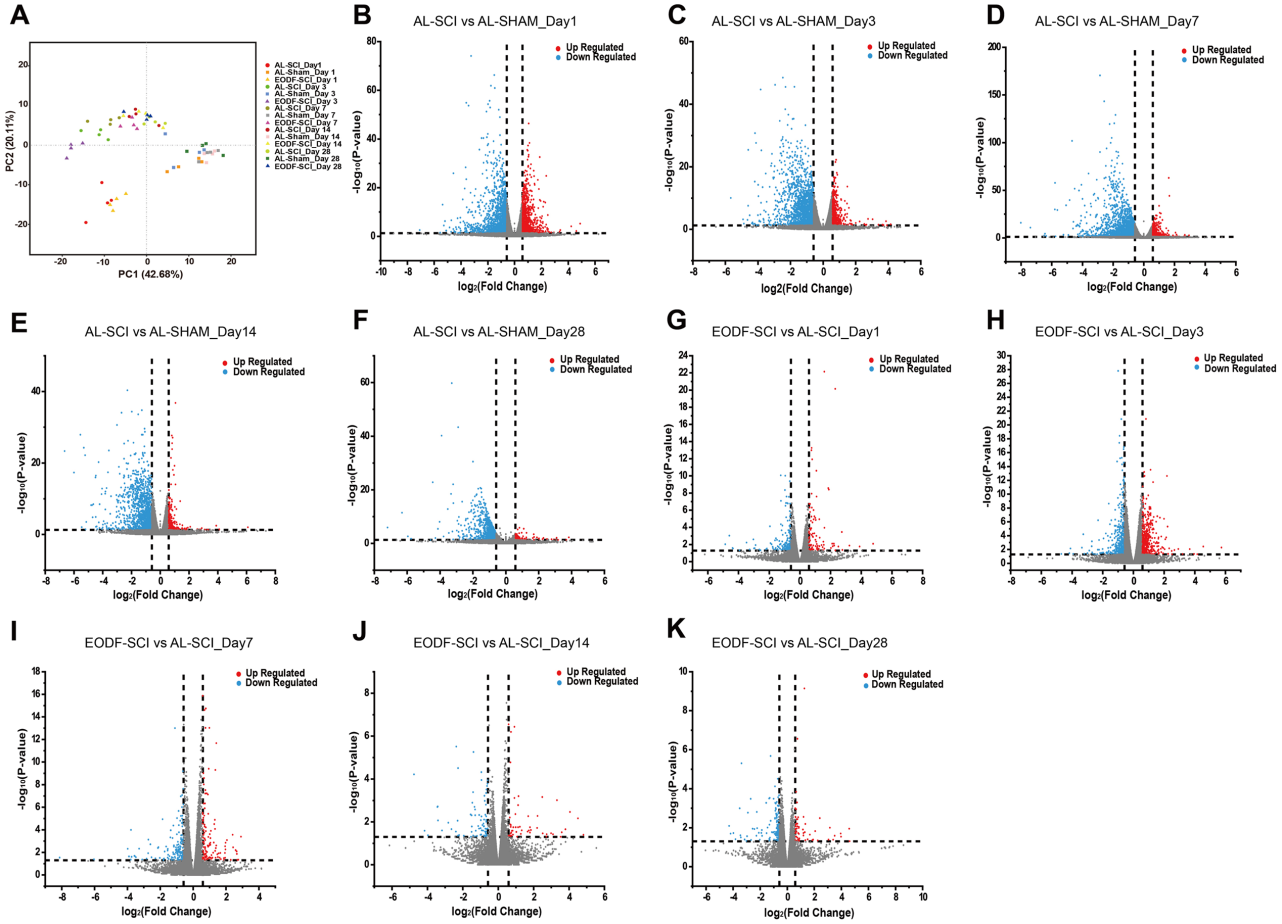
Gene transcriptome analysis of spinal cord tissue. **(A)** PCA scores of EODF-SCI, AL-SCI, and AL-SHAM gene counts at different time periods. **(B–F)** DEGs of AL-SHAM vs. AL-SCI in each time period (*p* < 0.05 and |log2(foldchange)| > 0.58). **(G–K)** DEGs of EODF-SCI vs. AL-SCI in each time period (*p* < 0.05 and |log2(foldchange)| > 0.58).

We completed the sequencing of 60 samples with the reference transcriptome and obtained 452.80 G of clean bases. The distribution of Q30 bases was from 95.19 to 96.62%, and the effective data volume of each sample ranged from 6.30–8.47 G, with an average GC content of 49.50%. Genomic matches were obtained for each sample by comparing the reads to the reference genome, with a 97.40–98.19% match rate. Based on the results of the comparison, a principal component analysis (PCA) was performed on the number of counts of each sample gene (Figure 6A). Comparing the AL-SHAM and AL-SCI groups, on day 1, 2,481 DEGs were significantly regulated, with 935 up-regulated and 1,546 down-regulated (Figure 6B); on day 3, 2055 DEGs were significantly regulated, with 457 up-regulated and 1,598 down-regulated (Figure 6C); on day 7, 2,306 DEGs were significantly regulated, with 501 up-regulated and 1805 down-regulated (Figure 6D); on day 14, 1,422 DEGs were significantly regulated, with 214 up-regulated and 1,208 down-regulated (Figure 6E); and on day 28, 914 DEGs were significantly regulated, with 93 up-regulated and 821 down-regulated (Figure 6F). Comparing the EODF-SCI and AL-SCI groups, on day 1, 250 DEGs were significantly regulated, with 99 up-regulated and 151 down-regulated (Figure 6G); on day 3, 769 DEGs were significantly regulated, with 401 up-regulated and 368 down-regulated (Figure 6H); on day 7, 291 DEGs were significantly regulated, with 123 up-regulated and 168 down-regulated (Figure 6I); on day 14, 128 DEGs were significantly regulated, with 53 up-regulated and 75 down-regulated (Figure 6J); and on day 28, 198 DEGs were significantly regulated, with 66 up-regulated and 132 down-regulated (Figure 6K). These results indicated that SCI modeling caused considerable differences in the transcriptome level in spinal cord tissue. EODF participated in regulating the transcriptome level in spinal cord tissue at different time points, and its trend was significantly different from that of the AL treatment.

### 3.6. Functional enrichment analysis of DEGs

**Figure 7:**
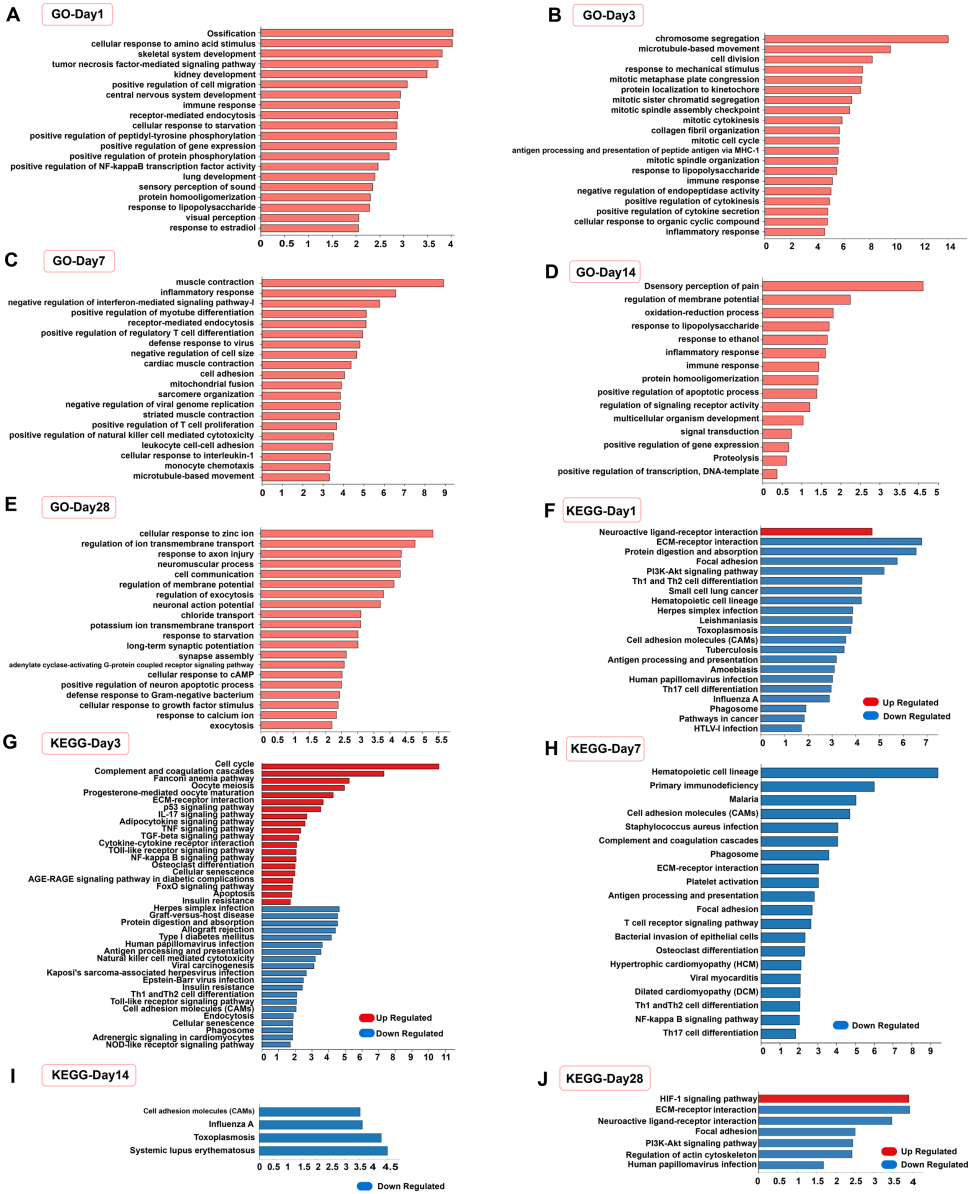
Functional analysis of differentially expressed genes. **(A–E)** Top 20 significantly enrichened GO terms in biological process (number of genes >3) of the EODF-SCI group versus the AL-SCI group at different time periods. **(F–J)** Top 20 significantly enriched KEGG pathways of up-regulated and down-regulated DEGs at different time periods.

The function of DEGs was assessed by GO and KEGG enrichment analyses, as demonstrated by the enrichment of the top 20 significant terms and pathways (number of genes >3 per entry). At 1, 3, 7, and 14 days after SCI, the DEGs in the EODF and AL groups were significantly altered by biological events, such as immune inflammatory responses, cell differentiation, and protein modifications (Figures 7A–D), For example, the GO terms “inflammatory response,” “immune response,” “tumour necrosis factor-mediated signal-ling pathway,” “positive regulation of NF-κB transcription factor activity,” “lipopolysaccharide response,” “positive regulation of regulatory T-cell differen-tiation” and “cellular response to interleukin-1” were all part of the immune inflammatory response; “positive regulation of cell migration,” “cell division,” “mitotic cell division,” “chromosome segregation,” “protein localization to kinetochores,” and “positive regulation of myotube differentiation” were all associated with cell differentiation; and “positive regulation of peptidyl tyrosine phosphorylation,” “positive regulation of protein phosphorylation,” “protein homologation” and “negative regulation of endopeptidase activity” were all related to protein modifications. At 28 days, biological events such as the immune inflammatory response were no longer the dominant biological events. In contrast, the terms “response to axon injury,” “synapse assembly,” “cellular response to growth factor stimulus,” and “positive regulation of neuron apoptotic process” suggested possible differences in neuronal differentiation (Figure 7E).

We the performed separate KEGG pathway enrichment analyses for up- and down-regulated DEGs and obtained similar results (Figures 7F–J). The pathways that were enriched at 1, 3, and 7 days after SCI were mainly associated with immune inflammatory responses, including “PI3K-Akt signaling pathway,” “NF-kappa B signaling pathway,” “P53 signaling pathway,” “Th1 and Th2 cell differentiation,” “Th17 cell differentiation,” “TNF signaling pathway,” “Toll-like receptor signaling pathway,” “NOD-like receptor signaling pathway,” and “FoxO signaling pathway.” In addition, at 1 and 3 days after SCI, DEGs were enriched in the “neuroactive ligand-receptor interaction” pathway, representing a collection of all receptors and ligands associated with intra- and extracellular signaling pathways on the plasma membrane. Active biological events occurred 3 days after SCI. In addition to immune inflammation-related pathways, entries in the “TGF-beta signaling pathway” and “apoptosis” were enriched, suggesting differences in biological events, such as nerve growth and apoptosis, after EODF intervention. At 14 days after SCI, DEGs were enriched in cell adhesion molecules, which is a pathway associated with cell adhesion. At 28 days after SCI, the “HIF-1 signaling pathway” was a stress signaling pathway for organisms in a hypoxic state. These results suggested that EODF may have exerted neuroprotective effects by modulating multiple biological events during a 28-day treatment cycle in SCI rats.

### 3.7. DEGs associated with neuroprotection

**Figure 8:**
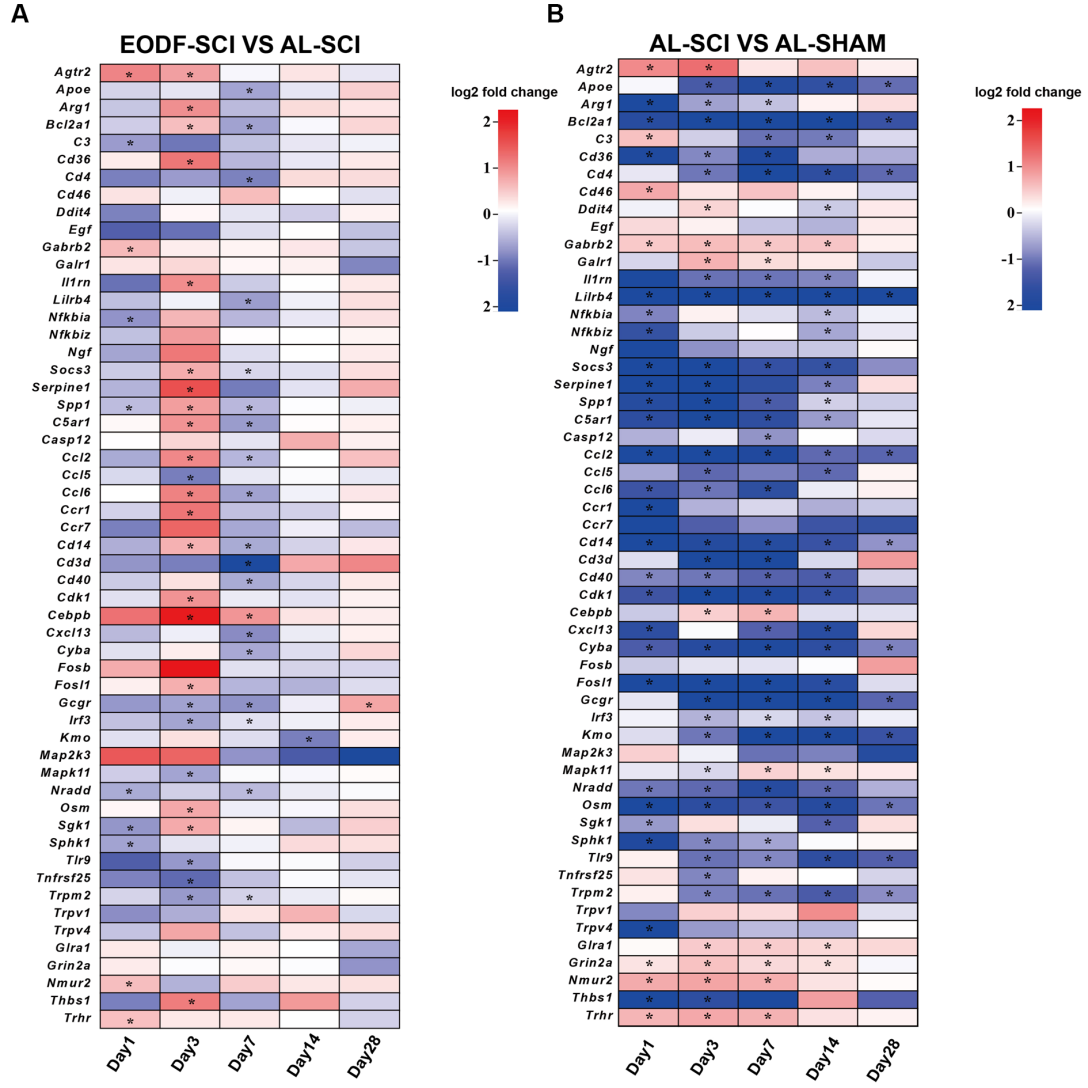
DEGs associated with neuroprotection. DEGs associated with neuroprotective effects between: **(A)** the EODF-SCI and AL-SCI groups; and **(B)** the AL-SCI and AL-SHAM groups. log2(foldchange) > 0 indicates up-regulated DEGs, and log2(foldchange) < 0 indicates down-regulated DEGs; *p < 0.05.

The ability of EODF to exert neuroprotective effects in SCI rats was been demonstrated; thus, we sought to determine how EODF regulated gene levels (closely associated with neuroprotection) in SCI rats at 1, 3, 7, 14, and 28 days. Therefore, we further performed an in-depth analysis of the GO and KEGG entries enriched in the EODF-SCI and AL-SCI groups that were associated with neuroprotection (Supplementary Table S2). We used the AL-SHAM group as a baseline, and the DEGs in the entries that were associated with neuroprotection in the EODF-SCI group versus the AL-SCI group are shown in Figures 8A,B, and mainly included: (i) genes with beneficial effects on neuroprotection that are associated with: (1) microglia polarization (*Socs3 and Arg1*); (2) anti-inflammatory effects (*Agtr2, Apoe, C3, Cd36, Gabrb2, Il1rn, Nfkbia,* and *Spp1*); (3) anti-apoptosis (*Bcl2a1*); and (4) nourishing nerves and promoting nerve repair (*Cd4 and Serpine1*); (ii) genes related to nerve injury: (1) pro-inflammatory (*Ccl5, Ccl6, Ccr1, Cebpb, Fosl1, Mapk11, Osm, Sphk1, Tnfrsf25, and Trpm2*); (2) pro-oxidative stress (*Cyba and Sgk1*); (3) pro-inflammatory and induced oxidative stress (*Tlr9*); (4) immune infiltration and activation (*Cd4,Cd14, Cd3d, Cd40, and Cxcl13*); (5) promotion of apoptosis (*C5ar1, Cdk1, Irf3, and Nradd*); and (6) other genes: neurotoxin production (*Kmo*), encodes neurexin U receptor (*Nmur2*) and unknown functional genes (*Thbs1* and *Trhr*). Our results revealed that DEGs associated with neuroprotective effects in both groups were mainly clustered at 1, 3, and 7 days after SCI. Compared to the Al-SCI group, the EODF-SCI group showed predominantly down-regulated DEGs at 1 days, predominantly up-regulated DEGs at 3 days, and predominantly down-regulated DEGs at 7 days. Two DEGs were observed at 14 and 28 days. The above results suggested that the expression levels of DEGs related to neuroprotection at different time points after EODF intervention in SCI rats were significantly different from those in the AL group both spatially and temporally, among which inflammation-related genes accounted for the majority. Therefore, we suggest that EODF may have played an important role in the regulation of inflammation in the acute and subacute phases in SCI rats.

### 3.8. Association between gut microbiota and neuroprotection-related DEGs

**Figure 9:**
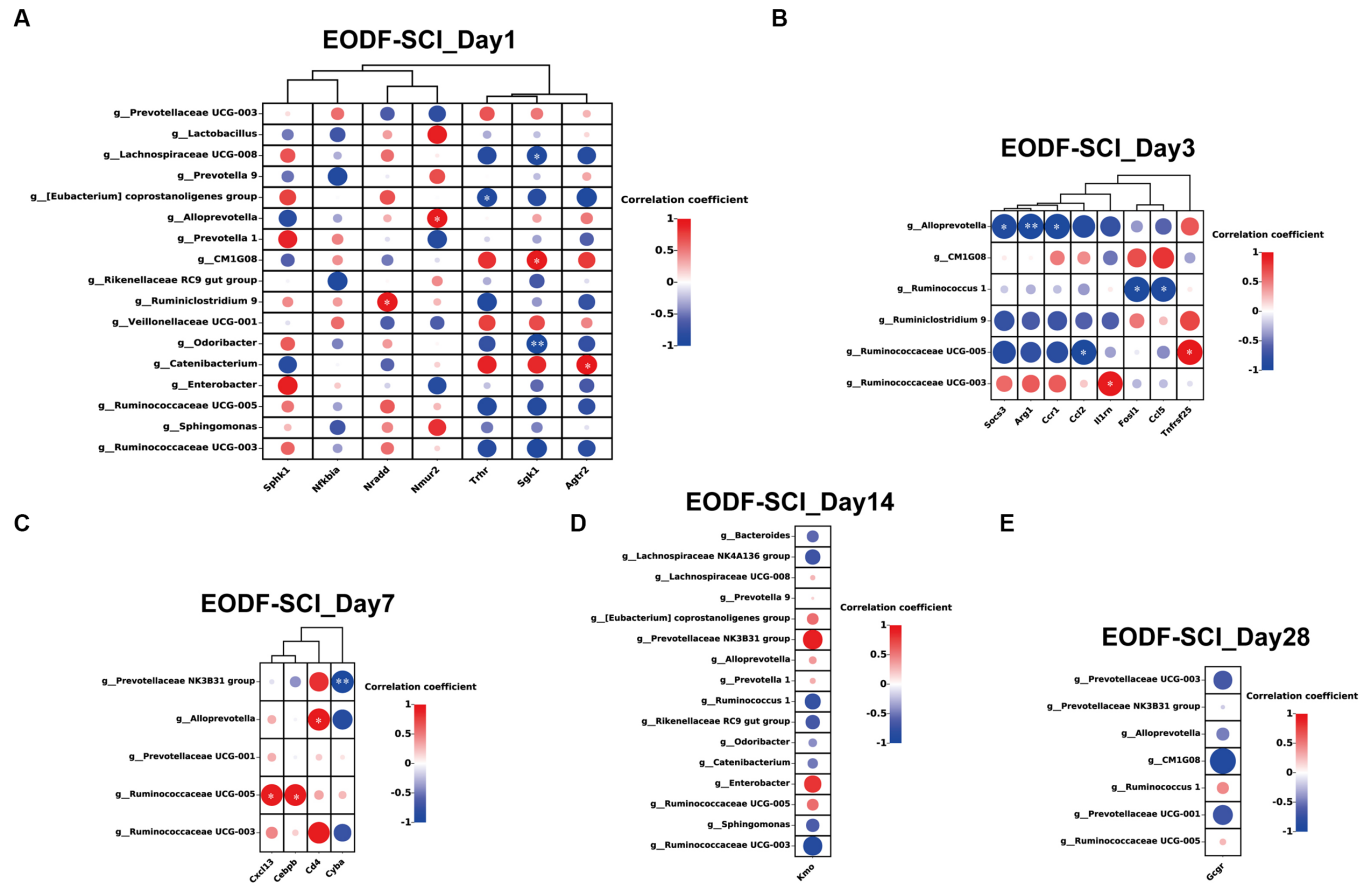
Association between gut microbiota (genera with significant differences from the AL-SCI group) and neuroprotection-related DEGs. **(A)** EODF-SCI_Day1; **(B)** EODF-SCI_Day3; **(C)** EODF-SCI_Day7; **(D)** EODF-SCI_Day14; and **(E)** EODF-SCI_Day28. The color gradient illustrates the Spearman’s rank correlation coefficient: red indicates a positive correlation and blue indicates a negative correlation. *p < 0.05, **p < 0.01.

Next, we performed Spearman’s correlation analysis of the abundance of gut microbiota in the EODF-SCI group (different genera from the AL-SCI group) with neuroprotection-related DEGs. On day 1, the anti-inflammatory gene *Agtr2* was significantly positively correlated with *Catenibacterium* (*r* = 0.985, *p* = 0.015); and the pro-inflammatory gene *Sgk1* was significantly positively correlated with *CM1G08* (*r* = 0.969, *p* = 0.031) and negatively correlated with *Lachnospiraceae UCG-008* (*r* = −0.955, *p* = 0.045) and *Odoribacter* (*r* = −0.992, *p* = 0.008). The pro-apoptotic gene *Nradd* was significantly positively correlated with *Ruminiclostridium 9* (*r* = 0.968, *p* = 0.032); *Nmur2* was significantly positively correlated with *Alloprevotella* (*r* = 0.958, *p* = 0.042); and *Trhr* was significantly negatively correlated with *[Eubacterium] coprostanoligenes group* (*r* = −0.950, *p* = 0.049; Figure 9A). On day 3, *Socs3* (*r* = −0.963, *p* = 0.037)*, Arg1* (*r* = −0.992, *p* = 0.008), and *Ccr1* (*r* = −0.981, *p* = 0.018) were significantly negatively correlated with *Alloprevotella*; *Ruminococcaceae UCG-005* was significantly negatively correlated with *Ccl2* (*r* = −0.976, *p* = 0.024) and significantly positively correlated with *Tnfrsf25* (*r* = 0.984, *p* = 0.016); *II1rn* was significantly positively correlated with *Ruminococcaceae UCG-003* (*r* = 0.959, *p* = 0.041); and *Fosl1* (*r* = −0.987, *p* = 0.013) and *Ccl5* (*r* = −0.967, *p* = 0.033) were significantly and negatively correlated with *Ruminococcus1* (Figure 9B). On day 7, *Ruminococcaceae UCG-005* was significantly positively correlated with *Cxcl13* (*r* = 0.953, *p* = 0.047)*, Cebpb* (*r* = 0.955, *p* = 0.045); *Alloprevotella* was significantly positively correlated with *Cd4* (*r* = 0.970, *p* = 0.030); and *Prevotellaceae NK3B31 grou*p was significantly negatively correlated with *Cyba* (*r* = −0.992, *p* = 0.008; Figure 9C). However, no genera of DEGs were found to be associated with neuroprotection on days 14 or 28 (Figures 9D,E). The above results suggested that EODF was able to reregulate the abundances of certain gut microbiota that are closely associated with neuroprotective-associated DEGs in SCI rats during the acute and subacute phases of SCI.

## 4. Discussion

EODF, as a dietary intervention therapy, could exert neuroprotective effects and facilitate functional recovery in spinal cord injured rats. We also confirmed that EODF intervention promoted motor function in rats with SCI (C5 half of the spinal cord). However, the effects of EODF treatment on the gut microbiota and the transcriptomic gene expression of spine cord tissues of SCI rats during the acute, subacute, and recovery periods were unknown; therefore, we assessed the long-term effects of EODF treatment on the dynamics changes in gut microbiota and transcriptomic gene expression of spine cord tissues of SCI rats by 16sDNA high-throughput sequencing and RNA transcriptome technology.

Gut microbiota diversity was significantly higher in healthy rats treated with EODF than that in the AL-treated group, based on the Simpson and Shannon indices. Similarly, a recent study found that gut microbiota α diversity was significantly higher in obese mice after a low-fat diet (LFD) combined with IF treatment than with the LFD combined with AL treatment (Deng et al., 2020). By analyzing the α-diversity at each time point in the three groups, our study showed that in the EODF-SCI group, the gut microbiota structure changed dynamically, the abundance of gut microbiota showed a trend of decreasing and then increasing, and its diversity showed a trend of decreasing, then increasing, then decreasing, and finally increasing. In the AL-SCI group, both the abundance and diversity of gut microbiota showed a trend of increasing and then remaining stable. In the AL-SHAM group, both the abundance and diversity of gut microbiota showed a trend of increasing, then decreasing, and finally increasing. We also found that on day 14, the abundance of gut microorganisms was significantly lower in the EODF-SCI group than in the AL-SCI group. On days 1, 7 and 28, the diversity of gut microbiota in the EODF-SCI group was significantly reduced. Analysis of β diversity analysis showed that in the EODF-SCI group, the structure of the gut microbiota differed on days 3 and 28 from that at the other time points; in the AL-SCI group, the structure of the gut microbiota differed from that at the other time points on days 1 and 28; in the AL-SHAM group, the structure of the gut microbiota differed from that at the other time points on days 14 and 28. In the SCI rat models, the most dominant phyla in the EODF-SCI, AL-SCI, and AL-SHAM groups were *Firmicutes* and *Bacteroidetes*. The *Firmicutes/Bacteroidetes* ratio was significantly lower in the EODF-SCI group on days 3 and 28 compared to that in the pre-modeling period (day 0), which differed from the changes in the AL-SCI and AL-SHAM groups. The *Firmicutes/Bacteroidetes* ratio was significantly higher in the EODF-SCI group on days 1, 7, and 14 and significantly lower on day 28 compared to those in the AL-SCI group. *Firmicutes* and *Bacteroidetes*, which account for ~90% of gut microbiota, have various important functions in host physiology (Rinninella et al., 2019). The *Firmicutes/Bacteroidetes* ratio is crucial for maintaining healthy intestinal homeostasis (Stojanov et al., 2020). In the rat model of SCI, by comparing the abundances of the top 10 ranked phylum level bacteria in the EODF-SCI group with those in the AL-SCI group, we found that the differential bacteria were mainly concentrated on days 1 and 14.

At the genus level, in the EODF group, with the exception of *Veillonellaceae UCG-001*, the abundances of the other bacterial populations changed dynamically at different time points. We analyzed the relative abundances and variations of potentially anti-inflammatory genera (including *Prevotella*, *Lactobacillus*, and *Lachnospiraceae*) and pro-inflammatory genera (*Bacteroides*), which showed dynamic changes in both the AL and EODF groups. On days 1 and 14 after SCI, there was a significant increase in the abundances of *Prevotella1, Prevotella9,* and *Lachnospiraceae UCG-008* in the EODF-SCI group compared to those in the AL-SCI group. Preclinical studies in models of rheumatoid arthritis (Marietta et al., 2016) and experimental acute encephalomyelitis (Mangalam et al., 2017; Shahi, 2019) have shown that oral treatment with this *Prevotella* strain (*P. histicola*) has immunomodulatory effects leading to reduced inflammation development and severity. EDP1815 is prepared from a single strain of *Prevotella* (*P. histicola*). Phase 2 dose-ranging studies in mild and moderate psoriasis [NCT04603027] and a phase 2 study in mild to severe atopic dermatitis [NCT05121480] have been conducted, and these results suggest that EDP1815 has the potential to be used in the treatment of a variety of inflammatory conditions, introducing a new class of drug to the medical community (Itano et al., 2023). *Lachnospiraceae* abundance was decreased in diarrheal children, and supplementing mice with *Lachnospiraceae* reduced obesity-related symptoms and inflammation (Truax et al., 2018; Castro-Mejía et al., 2020). Interestingly, in the present study, on day 1 after SCI, there was a significant increase in the *Lactobacillus* abundance in the EODF-SCI group compared to that in the AL-SCI group. *Lactobacilli* are the most common colonizing bacteria in the gastrointestinal tract of healthy humans and exert beneficial effects by regulating the gut microbiota (Goldstein and Hager, 2015). In addition, *Lactobacillus* exerted neuroprotective effects by rebuilding the microbiota in mice with traumatic brain injury (Ma et al., 2019). A previous study suggests that the *Lactobacillus* app. Cocktail has anti-inflammatory effects on HT-29 cells by modulating the JAK/STAT and NF-κB signaling pathways and that *Lactobacillus* as a dietary supplement may prevent and reduce inflammation-related diseases (Aghamohammad et al., 2022). Anukam et al. found that the *Lactobacillus rhamnosus* and *Lactobacillus reuteri* may help to down-regulate urinary tract infection-related inflammatory factors in patients with SCI (Anukam et al., 2009); in the present study, all five time points after SCI modeling in the EODF-SCI group showed significant reduction in the *Alloprevotella* abundance compared to that in the AL-SCI group. The biological function of *Alloprevotella* is unknown; however, one study suggested that ginkgolide B (GB) is neuroprotective and that GB treatment may exert a neuroprotective effect in AD mice by increasing the *Lactobacillus* abundance and decreasing the abundances of *Alloprevotella, Bacteroidales,* and *Muribaculaceae* (Liu et al., 2021b). On day 14 after SCI, there was a significant decrease in the *Bacteroide* abundance in the EODF-SCI group compared to that in the AL-SCI group. Several studies have suggested that *Bacteroides* have potential pro-inflammatory properties (Lindberg et al., 1990; Zhang et al., 2010; Gomez et al., 2012; Munyaka et al., 2016; Ryan et al., 2020). These findings suggested that remodeling of the gut microbiota by EODF treatment may have been beneficial in SCI rats. Particularly on day 1 after SCI in rats (defined as the acute phase), there was a significant increase in the abundances of anti-inflammatory genera in the EODF-SCI group compared to those in the AL-SCI group. We suspect that these beneficial flora may have exerted an anti-inflammatory effect in the acute phase of SCI in rats, and since probiotic products such as *Lactobacillus.app* and *Prevotella.app* are available for purchase, this presents a promising outlook. These probiotics alone, in combination, and in a cocktail, in future studies.

A succession of pathophysiological processes that commence minutes after the original trauma and occur in addition to the primary damage constitute secondary injuries following SCI. They are divided into three phases: acute, subacute, and chronic (Ahuja et al., 2017; Alizadeh et al., 2019; Anjum et al., 2020; Li M. et al., 2022). In humans, the change from the acute to subacute phase often takes place between a few hours and 48 h following the injury. The transition from the acute to the chronic phase, however, is anticipated to take place within 6 months (Ahuja et al., 2017). In contrast, there are three phases of secondary damage in rodents: acute (lasting <24 h), subacute (lasting between 24 h and 7 days), and chronic (lasting >7 days; Li M. et al., 2022). Our transcriptomic data analysis revealed that on days 1, 3, 7, and 14 days after SCI, DEGs between the EODF and AL groups were significantly enriched in entries related to biological events, such as immune response, inflammatory response, cell differentiation, and protein modification. This analysis also showed that at 28 days, entries such as immune and inflammatory responses were no longer significantly enriched, with significantly enriched entries suggesting that major biological events may be associated with neuronal cell differentiation. The results of the KEGG pathway enrichment analysis indicated that the pathways enriched in the EODF and AL groups with DEGs at 1, 3, and 7 days after SCI were mainly associated with immune and inflammatory responses. The studies pointed out that many biological events are involved in different stages of injury after SCI; In the acute phase, the main biological events are associated with immune cell activation, inflammatory response, vascular damage, and oxidative damage;the primary biological occurrences in the subacute period are linked to glial scar formation, reactive astrocyte activation, axonal remodeling, and neuronal death (Alizadeh et al., 2019; Pinchi et al., 2019; Anjum et al., 2020; Zhang et al., 2021). In the acute phase after SCI, the main biological events are associated with neutrophils and microglia, which are immediately activated and secrete inflammatory factors such as interleukin-1β (IL-1β), tumor necrosis factor-alpha (TNF-α) and interleukin 6 (IL-6). The primary biological activities that occur during the subacute phase are linked with monocytes and macrophages entering the spinal cord and releasing pro-inflammatory cytokines, chemokines, and other inflammatory mediators. In both the subacute and chronic stages, the neuroinflammatory response is the response that ultimately causes cell death and tissue degradation, making it an essential component of secondary damage (Hellenbrand et al., 2021). Therefore, we hypothesize that EODF exerts its neuroprotective effects mainly by modulating the immune response and inflammatory response pathways in the early (acute or subacute) phase of SCI.

We further screened the DEGs associated with neuroprotective effects in the AL-SCI and EODF-SCI groups on days 1, 3, 7, 14, 28 days after SCI. and these DEGs were mainly concentrated on days 1, 3, and 7. This further suggested that EODF treatment was able to modulate neuroprotective effects related to DEGs, and that its modulation time was mainly focused on the acute and subacute phases after SCI. These included genes that exert beneficial effects on neuroprotection: (1) *Socs3* and *Arg1* are associated with microglia polarization, with *Socs3* potentially inhibiting microglia polarization and thereby exerting a neuroprotective effect (Wu et al., 2016; Aliena-Valero et al., 2021); *Arg-1* expression presents an association with the M2 phenotype in microglia, and M2-type microglia are considered a beneficial cell type for recovery from neural injury (Li et al., 2021); (2) *Agtr2, Apoe, C3, Cd36, Gabrb2, Il1rn, Nfkbia, and Spp1*, which express proteins thought to be involved in exerting anti-inflammatory effects (Marie et al., 2002; Tukhovskaya et al., 2010; Yeung et al., 2018; Huang et al., 2020; Kisucká et al., 2021; Hou et al., 2022; Kaltschmidt et al., 2022; Li and Jakobs, 2022; Shen et al., 2022); (3) *Bcl2a1*, which exert anti-apoptotic effects (Bedirli et al., 2018); and (4) *Cd4 and Serpine1*, which are involved in nourishing and promote nerve repair (Calenda et al., 2012; Kofke et al., 2018). (i) The DEGs also included genes involved in nerve injury: (1) *Ccl5, Ccl6, Ccr1, Cebpb, Fosl1, Mapk11, Osm, Sphk1, Tnfrsf25,* and *Trpm2*, for which the associated proteins may have been involved in exerting pro-inflammatory effects, including those involved in activating the NF-κB signaling pathway, PI3 kinase/Akt pathway, and CCR1/TPR1/ERK1/2 signaling pathway (Kanno et al., 2005; Li J. et al., 2020; Li M. et al., 2020; Yan et al., 2020; Komori et al., 2022); (2) *Cyba* and *Sgk1*, which promote oxidative stress (Iqbal et al., 2015; Usategui-Martín et al., 2022); (3) *Tlr9*, which are involved in both pro-inflammatory and induction of oxidative stress, and signal activation, and potentially exacerbate neurodegeneration by inducing oxidative stress and inflammation (Dalpke et al., 2002); (4) *Cd4, Cd14, Cd3d, Cd40,* and *Cxcl13*, which promote immune infiltration and activation (Krumbholz et al., 2006; Hernandez et al., 2007; Wang et al., 2022; Daniel et al., 2023) and (5) *C5ar1, Cdk1, Irf3,* and *Nradd*, which promote apoptosis (Wang et al., 2003; Canudas et al., 2004; Solis et al., 2006; Cui et al., 2016; Ding et al., 2021; Shi Y. et al., 2021). (ii) The DEGs also included other genes: (1) Kmo, which expresses proteins that increase the production of toxic kynurenine pathway metabolites in the brain production, such as the neurotoxins 3-hydroxykynurenine and quinolinic acid, and decrease the neuroprotective metabolite kynurenic acid (Chen et al., 2022). Our results revealed that DEGs associated with neuroprotective effects in both groups were mainly clustered on days 1, 3, and 7 after SCI; among them, DEGs related to inflammation accounted for the majority, and the expression levels of DEGs related to neuroprotection were significantly different both temporally and spatially from those in the AL group at different time points after EODF intervention in SCI rats. These results also suggested that EODF may have played an important role in the regulation of immune–inflammation in the acute and subacute phases of SCI rats.

Interestingly, among the DGEs involved in neuroprotection-related genes screened in this study, *Cebpb*, *Socs3*, *Arg1*, *Apoe*, *C3*, *Ccl2,* and *Ccl3* were strongly associated with fasting. Goldstein et al. (2017) found by measuring chromatin accessibility that fasting majorly reorganizes liver chromatin, exposing many fasting-induced enhancers, among which *Cebpb* is one of the key transcription factors regulating the fasting response. c/EBPs are considered constitutive transcription factors, and fasting-related signals glucocorticoids and glucagon increase the expression and activity of C/EBPβ (the protein encoded by Cebpb), and reducing C/EBPα levels through chromatin regulation impairs gluconeogenesis (Goldstein et al., 2015). In addition, *Socs3* (García et al., 2010; Vendelbo et al., 2015) and *Arg1* (Dai et al., 2022), which have neuroprotective benefits, had increased expression levels under the influence of fasting. *Apoe* is the main apolipoprotein synthesized in the brain in response to injury (Tukhovskaya et al., 2010; Mérian et al., 2023) showed that knocking out *Apoe* increased the susceptibility of mice to cardiovascular disease, and the IF improved glycolipid metabolism in *Apoe*−/− mice. Yamamoto et al. found increased complement C3 levels in a baseline model of cardiac ischemia–reperfusion mice and decreased complement C3 levels in the calorie-restricted group compared to the baseline group (Yamamoto et al., 2016). Liu et al. (2019) performed an 8-week dietary intervention in mice and found that in mice on an IF regime (i.e., continuous fasting for 24 h 3 days per week) IF decreased mRNA levels of macrophage markers (Ccl2 and Ccl3) in inguinal and gonadal adipose and reduced adipose tissue macrophage numbers compared to those in mice on a high-fat diet (HFD, 43% fat). Therefore, the above genes are associated with fasting either by playing a role in the fasting process or as a result of fasting intervention.

**Figure 10:**
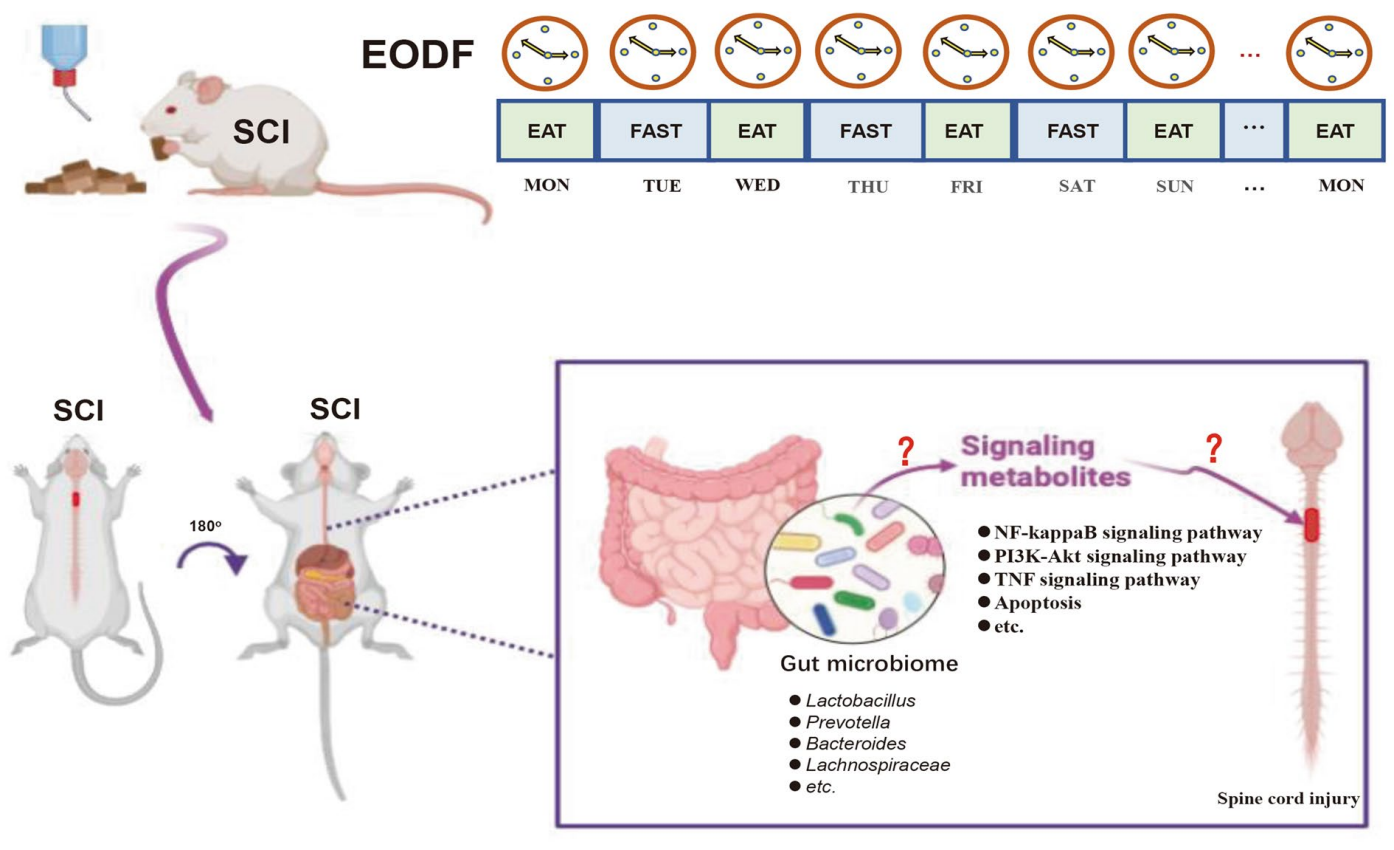
Hypothetical model of the mechanism by which EODF-mediated gut microbiota remodeling exerted neuroprotective effects in SCI rats.

Finally, we performed a Spearman’s correlation analysis of the DEGs associated with neuroprotection at the gut microbiota abundances (differential genera with those of the AL-SCI group) in the EODF-SCI group. Our results found that EODF was able to regulate the abundance of certain gut microbiota that are closely associated with neuroprotective-associated DEGs in SCI rats during the acute and subacute phases of SCI. Gut microbiota has been shown to play a neuroprotective role in various neurological disorders. Gut microbiota attenuates oxidative stress and inflammatory aspects through its metabolites or the production of secondary metabolites. Moreover, modulation of the gut microbiota with antioxidant and anti-inflammatory probiotics has also shown promising neuroprotective effects (Zhu et al., 2018; Shruti et al., 2022; Solch et al., 2022). Based on this, we hypothesized that the remodeling of the gut microbiota by EODF conferred a neuroprotective effect on the rat model of SCI (Figure 10).

EODF can exert neuroprotective effects and facilitate functional recovery in SCI rats. To allow clinical translation of EODF, we previously conducted a safety study of EODF in patients with SCI (Zheng et al., 2021), and although most of the subjects completed this clinical study, it was found through a post-survey follow-up that this dietary intervention required patients to endure hunger on fasting days, and that many SCI patients and families were less willing to attempt it again. We have also contemplated whether the gut microbiota after EODF intervention could be used as a clinical alternative to EODF. Probiotics have been used to treat urinary tract infections and gastrointestinal discomfort in patients with SCI (Wong et al., 2014). In an animal study, intervention with VSL#3, a medical grade probiotic, was found to improve immune function and promote recovery of motor function in mice with SCI (Laginha et al., 2016). In addition to probiotic intervention, the fecal microbiota transplantation (FMT) approach was also effective in improving motor function and anxiety-like behavior after SCI (Schmidt et al., 2020; Jing et al., 2021, 2022). A recent study demonstrated that EODF intervention can effectively promote axonal regeneration after sciatic nerve compression, and that EODF can exert neuroprotective effects in a mouse model of sciatic nerve injury by means of FMT (Serger et al., 2022). Finally, EODF was found to promote the production of indole-3-propionic acid and affect the metabolism of the organism by increasing the abundance of gut gram-positive *Clostridiales* bacteria, thus achieving the effects of promoting axonal regeneration and accelerating the recovery of sensory function (Serger et al., 2022). Interestingly, They found that both *Clostridiales* bacteria and indole-3-propionic acid supplementation were able to exert similar therapeutic effects as those of EODF. Based on the above study, whether or not the gut microbiota after EODF intervention can protect AL-SCI rats is a very important and clinically translatable scientific question that deserves further study. In the future, the mediating role of gut microbiota can be verified by transplanting the healthy rat flora (EODF intervention) into AL-SCI rats by FMT. It is also necessary to conduct an in-depth investigation of the candidate bacteria that are closely related to neuroprotection after EODF intervention to verify whether they can exert similar effects to those of EODF. The findings of our study provide a further basis for treating SCI by targeting the gut microbiota.

## 5. Conclusion

In this study, we found that EODF could alter the abundance and diversity of the gut microbiota in SCI rats at different time points. Moreover, we revealed characteristic changes in the gut microbiota at various phylogenetic levels. Notably, EODF induced dynamic changes in the abundance of potentially anti-inflammatory and pro-inflammatory bacteria. In addition, spinal cord transcriptome sequencing analysis revealed that: (i) enrichments of DEGs at different time points after EODF intervention in SCI rats compared to that after AL intervention were associated with biological events, such as immune response, inflammatory response, cell differentiation, protein modification, apoptosis, and nerve cell differentiation; (ii) EODF was involved in regulating the levels of DEGs associated with neuroprotection after the onset of SCI, and this expression level was significantly different both spatially and temporally from that of the AL group; (iii) a significant correlation was observed between the relative abundance of certain genera and DEGs associated with neuroprotective effects in spinal cord tissues at different times points in the EODF-SCI group. Although further research is required to elucidate the mechanism underlying the neuroprotective effect of the EODF treatment in SCI rats, by combining the gut microbiota and transcriptome analyses results, we propose that gut microbiota may have been involved in mediating EODF to regulate the transcriptional levels of genes associated with neuroprotective effect in SCI rats, thereby exerting a neuroprotective effect. It should be noted that our study only analyzed the gut microbiota and tissue transcriptome levels. In future studies, a multiomics combination is needed to further explore the specific mechanisms of the neuroprotective effects of EODF in SCI treatment, such as through the combined application of proteomics, metabolomics, single-cell sequencing technology, and genetic engineering.

## Data availability statement

The raw sequence data reported in this paper have been deposited in the Genome Sequence Archive (Genomics, Proteomics & Bioinformatics 2021) in National Genomics Data Center (Nucleic Acids Res 2022), China National Center for Bioinformation / Beijing Institute of Genomics, Chinese Academy of Sciences (GSA: CRA011825 and CRA011760) that are publicly accessible at https://ngdc.cncb.ac.cn/gsa.

## Ethics statement

The animal study was reviewed and approved by the Western Theater General Hospital Animal Experimentation Ethics Committee (approval number: 2019ky79).

## Author contributions

AZ, RP, and JW conceived and designed the study. JW, XZ, RZ, MW, WX, ML, and RT performed the experiments. RP, JW, XZ, RZ, and MW interpreted the results of experiments. JW, XZ, RZ, and MW analyzed the data. JW, RZ, MW, and ZY prepared the figures. JW, RZ, and MW wrote the manuscript. JW, XZ, RZ, MW, JZ, JL, LZ, JG, and HL conducted the literature research. All authors contributed to the article and approved the submitted version.

## Funding

This work was supported by the General Program of the National Natural Science Foundation of China (81973927), the Key R&D Program of the Sichuan Department of Science and Technology (2021YFS0133), Sichuan Provincial Administration of Traditional Chinese Medicine Special Research Project on Traditional Chinese Medicine (2020LC0224), and the 2019 Annual Hospital Highland Medicine Research Project (2019ZY03). Projects on the science and technology situation in Tongliang District, Chongqing (2022005, TL2021-06).

## Conflict of interest

The authors declare that the research was conducted in the absence of any commercial or financial relationships that could be construed as potential conflicts of interest.

## Publisher’s note

All claims expressed in this article are solely those of the authors and do not necessarily represent those of their affiliated organizations, or those of the publisher, the editors and the reviewers. Any product that may be evaluated in this article, or claim that may be made by its manufacturer, is not guaranteed or endorsed by the publisher.

## Footnotes

^1^ http://drive5.com/uparse

^2^ http://geneontology.org/

^3^ http://www.genome.jp/kegg/

^4^ https://www.chiplot.online/
