# Supplementary material for: Gut microbiota and transcriptome dynamics in every-other-day fasting are associated with neuroprotection in rats with spinal cord injury
Source: Front Microbiol. 2023 Jul 28;14:1206909. doi: 10.3389/fmicb.2023.1206909 (PMC10417830; doi:10.3389/fmicb.2023.1206909)
Supplement: Supplementary file 1 [file Data_Sheet_1.pdf]

frontiers

Supplementary Material

Gut Microbiota and Transcriptome Dynamics in Every-Other-Day Fasting are Associated with Neuroprotection in Rats with Spinal Cord Injury

Junyu Wang [1†], Xiaohua Zhao [2,3†], Ruihan Zhou [2†],Meiyu Wang [4†], Wu Xiang[2], Ming Li[3], Runling Tang[3],Zilong You [5], Jingqi Zheng [2], Jiayu Li [6], Li Zhu [6], Jiaxin Gao [6],Huaqiang Li [6],Rizhao Pang [2*], Anren Zhang [7*]

[1] State Key Laboratory of Biotherapy, West China Hospital, Sichuan University, Chengdu 610041, China

[2] Department of Rehabilitation Medicine, General Hospital of Western Theater Command, Chengdu 610083, China

[3] Department of Rehabilitation Medicine, The People's Hospital of Tongliang District, Chongqing 402560, China

[4] Rehabilitation and Wellness Care Centre, Tian Fu College of Swufe, Chengdu 610083, China

[5] Department of Biochemistry and Biophysics, School of Basic Medical Sciences, Peking University, Beijing 100191, China

[6] School of Health Preservation and Rehabilitation, Chengdu University of Traditional Chinese Medicine, Chengdu 610075, China;

[7] Department of Rehabilitation Medicine, Shanghai Fourth People's Hospital Affiliated to Tongji University School of Medicine, Shanghai 200434, China

† These authors contributed equally to this work

***Correspondence:** przprz17@126.com (R.P.); anren0124@163.com (A.Z.)

1 Supplementary Figures and Tables

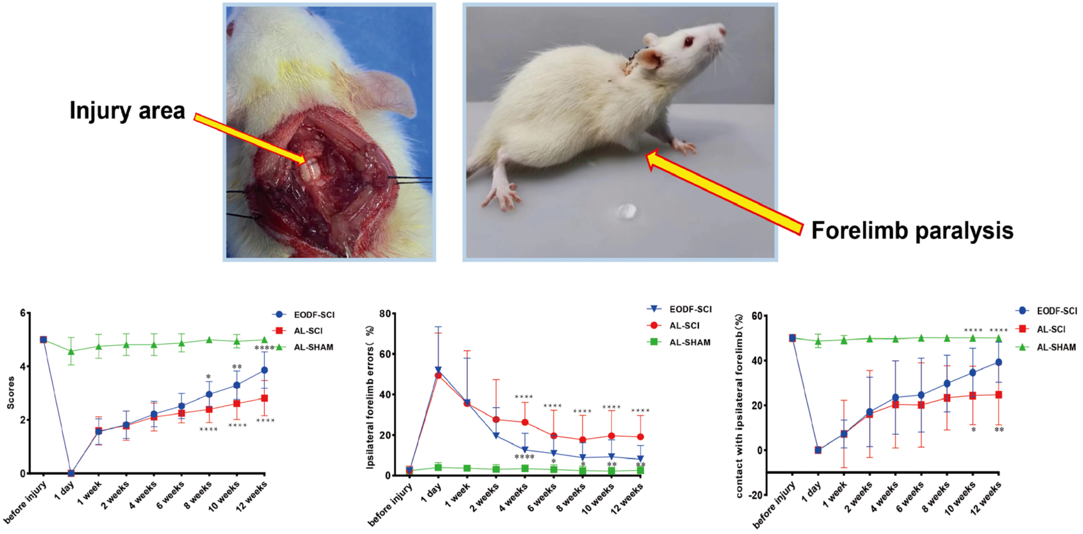

Supplementary Figure 1. Rat model of spinal cord injury (SCI) and the effect of EODF treatment on locomotor recovery. (A) C5 unilateral SCI by clamping and ipsilateral forelimb paralysis.(B-D) Time course of locomotor functional recovery as assessed by grooming test,Horizontal ladder test and Cylinder rearing test. ****p < 0.0001 indicates AL-SCI group compared to Sham group; *p < 0.05, **p < 0.01, ****p < 0.0001 indicates EODF-SCI group compared to AL-SCI group.

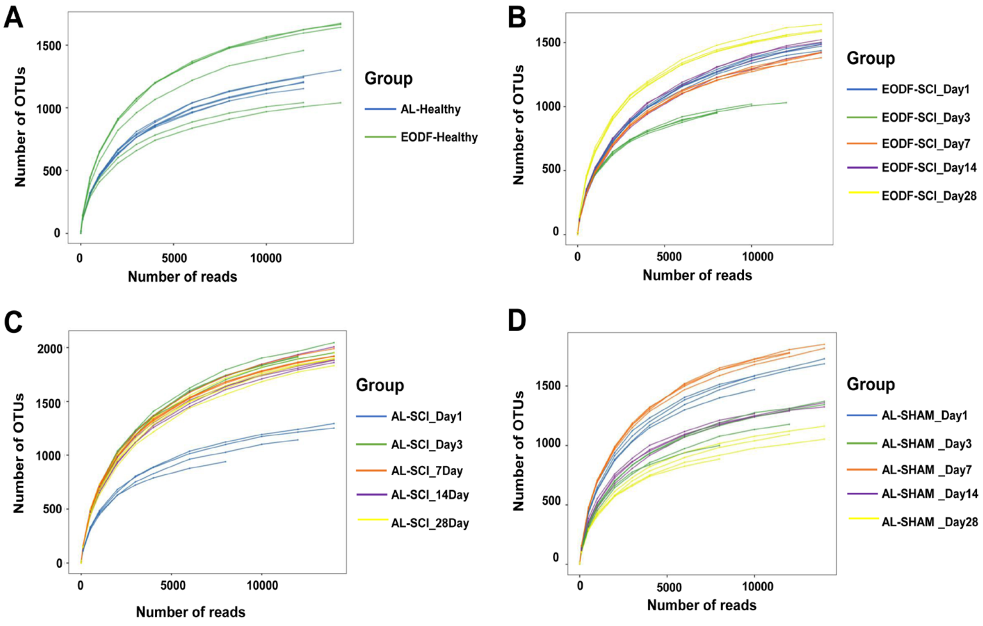

Supplementary Figure 2. Dilution curves of fecal samples. (A) Release profile of ad libitum diet (AL)-Healthy and every-other-day fasting (EODF)-Healthy groups. (B) Release curves of the EODF-SCI group at different time points. (C) Release curves of the AL-SCI group at different time points. (D) Release curves of the AL-SHAM group at different time points.

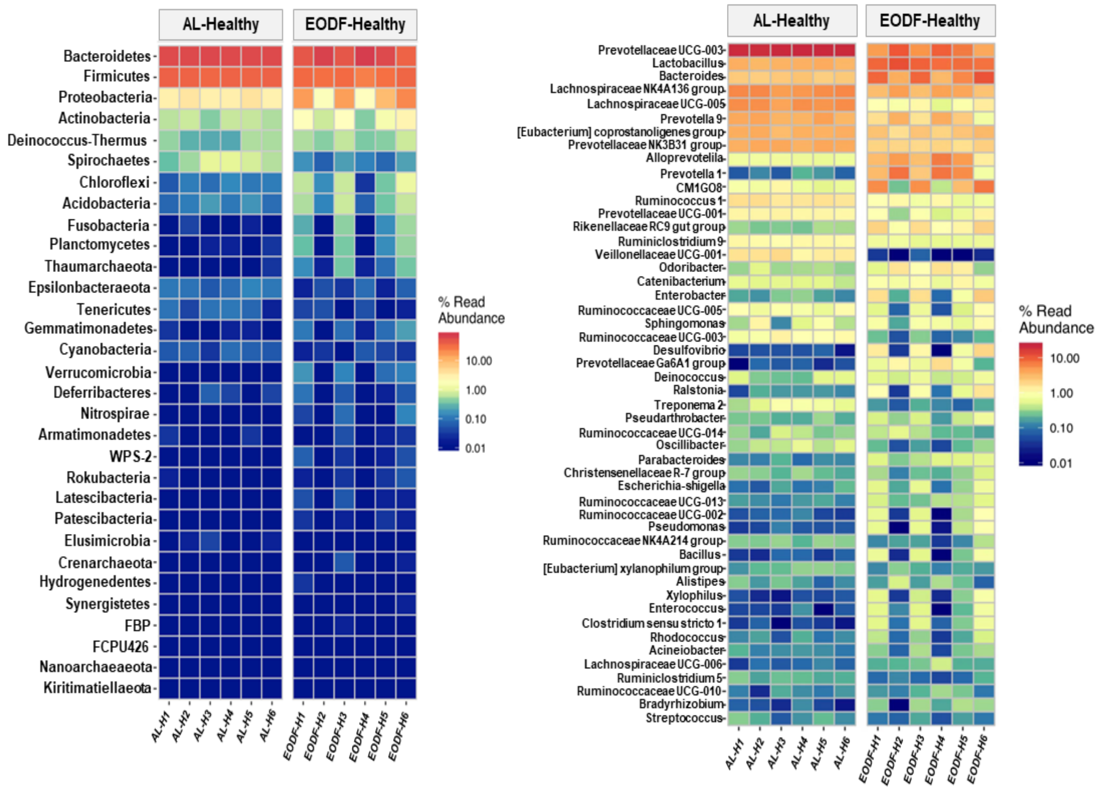

Supplementary Figure3. Characteristics of gut microbiota phyla and genera between the AL-Healthy and EODF-Healthy group. (A) Comparison of the phylum level composition of gut microbiota for each sample between groups (n = 6 per group). (B) Comparison of the genus-level gut microbiota composition for each sample between groups (n = 6).

Table S1 Comparison of genus levels between the AL-Healthy and EODF-Healthy groups.

Genus	AL-Healthy	EODF-Healthy	*P value*
Prevotellaceae UCG-003	21.04	6.70	0.000
Lactobacillus	3.43	8.82	0.000
Bacteroides	2.36	6.82	0.017
Lachnospiraceae NK4A136 group	5.57	3.57	0.002
Lachnospiraceae UCG-008	5.34	0.97	0.000
Prevotella 9	3.74	2.26	0.018
[Eubacterium] coprostanoligenes group	3.51	2.41	0.001
Prevotellaceae NK3B31 group	3.79	1.93	0.000
Alloprevotella	0.68	3.94	0.010
Prevotella 1	0.12	4.47	0.013
CM1G08	0.77	3.68	0.062
Ruminococcus 1	1.59	0.90	0.000
Prevotellaceae UCG-001	1.21	0.77	0.028
Rikenellaceae RC9 gut group	0.30	1.50	0.002
Ruminiclostridium 9	1.14	0.64	0.000
Veillonellaceae UCG-001	1.51	0.02	0.000
Odoribacter	0.35	1.05	0.024
Catenibacterium	0.50	0.89	0.006
Enterobacter	0.20	1.13	0.052
Ruminococcaceae UCG-005	0.86	0.47	0.046
Sphingomonas	0.58	0.72	0.512
Ruminococcaceae UCG-003	1.00	0.17	0.000

Table S2-GO enrichment analysis associated with neuroprotection			
1 Day	GO:0033209	tumor necrosis factor-mediated signaling pathway	Nfkbia; Tnfrsf25; Nradd; Card14
	GO:0006955	immune response	RT1-DOb; RT1-Da; Sbspon; Tnfrsf25; Nradd; Nfil3; Tlr9; LOC108348072
	GO:0051092	positive regulation of NF-kappaB transcription factor activity	Ins2; Sphk1; Tlr9; Cth; Card14
	GO:0032496	response to lipopolysaccharide	Hpgd; Acp5; Nfkbia; Tnfrsf25; Entpd1; Nradd; Slc11a1; Cebpb
3 Day	GO:0002474	antigen processing and presentation of peptide antigen via MHC class I	RT1-A1; RT1-A2; LOC100364500; RT1-CE4; RT1-M6-1; RT1-CE10
	GO:0032496	response to lipopolysaccharide	Lbp; Ptger1; Ptger3; Socs3; Dcn; Arg1; Ngf; Dio2; Nfkbia; Pawr; Tnfrsf25; Irf3; Ccl2; Ccl5; Trpv4; Junb; Ccr7; Ptgs2; Cd14; F3; Hmgb2; Ltc4s; C5ar1; Cebpb; Il1rn; Serpine1
	GO:0006955	immune response	Osm; RT1-A1; RT1-A2; Lat; LOC103690020; Sectm1b; LOC100364500; RGD1565355; RT1-CE4; Oas1b; Oas1g; Ms4a2; Tnfrsf25; Thbs1; Ccr1; Ccr7; Prg4; Cd36; Enpp3; Tlr9; Il1rn; RT1-CE10
	GO:0006954	inflammatory response	Lat; Ptger1; Ptger3; Agtr2; Ngf; Nfkbiz; Agtr1a; Kng1; Ms4a2; Tnfrsf25; Thbs1; Ccl2; Ccl5; Ccl6; Ccr1; Ccr7; Ptgs2; Ager; Cd14; C3; Tlr8; Igfbp4; C5ar1; Il1rn; Spp1
7 Day	GO:0006954	inflammatory response	Calca; Selp; Ccrl2; Cxcl13; Ccl6; Sdc1; Ucn; LOC681410; Cd14; Cd6; Cd40; Hck; Cd8a; Cela1; Cyba; C5ar1

	GO:0045591	positive regulation of regulatory T-cell differentiation	Pirb; Cd46; Lilrb4
	GO:0042102	positive regulation of T cell proliferation	Pirb; Il21; Cd4; Cd6; Cd46
	GO:0045954	positive regulation of natural killer cell-mediated cytotoxicity	Slamf6; Klrk1; Il21
	GO:0007159	leukocyte cell-cell adhesion	Calca; Selp; Itga5
	GO:0071347	cellular response to interleukin-1	Apoe; Adamts7; Mylk3; Ccl6; Cd40; Cebpb
	GO:0002548	monocyte chemotaxis	Calca; Folr2; Ccl6
14 Day	GO:0055114	oxidation-reduction process	Hpgd; Kmo; LOC103693015; Cyp4b1; Akr1b7; Tyrp1; LOC108348083
	GO:0032496	response to lipopolysaccharide	Hpgd; Pf4; Kmo; Gnrh1
	GO:0006954	inflammatory response	Pf4; Scn9a; Trpv1; Map2k3
	GO:0006955	immune response	RT1-DMa; RT1-DMb; Pf4
	GO:0043065	positive regulation of the apoptotic process	Hpgd; Clip3; Casp12; Trpv1
28 Day	GO:0048678	response to axon injury	Musk; Map1b; Ltc4s; Tspo
	GO:0060291	long-term synaptic potentiation	Tnr; Musk; Grin2a
	GO:0007416	synapse assembly	Pcdhb2; Pclo; Map1b
	GO:0007189	adenylate cyclase-activating G-protein coupled receptor signaling pathway	Gcgr; Galr1; Gpr26
	GO:0071320	cellular response to cAMP	Hcn1; Serpina3n; Serpine1
	GO:0043525	positive regulation of neuron apoptotic process	Musk; Itga1; Kcnma1
	GO:0071363	cellular response to growth factor stimulus	Grin2a; Map1b; Serpine1
Table S4-KEGG enrichment analysis			
1 Day	rno04080	Neuroactive ligand-receptor interaction	Agtr2; Gabrb2; Hcrtr2; Nmur2; RGD1560455; Trhr

	rno04151	PI3K-Akt signaling pathway	Col1a2; Col2a1; Col4a1; Col9a3; Ddit4; Egf; Ins2; Itga2b; Lama1; Lamb1; Sgk1
	rno04658	Th1 and Th2 cell differentiation	Cd4; Dll4; Nfkbia; RT1-DOb; RT1-Da
	rno04514	Cell adhesion molecules (CAMs)	Cd22; Cd4; Cldn19; Mpz; RT1-DOb; RT1-Da
	rno04659	Th17 cell differentiation	Cd4; Nfkbia; RT1-DOb; RT1-Da
3 Day	rno04115	p53 signaling pathway	Ccnb1; Ccnb2; Cdk1; Rrm2; Serpine1; Thbs1
	rno04657	IL-17 signaling pathway	Ccl2; Cebpb; Fosb; Fosl1; Nfkbia; Ptgs2
	rno04668	TNF signaling pathway	Ccl2; Cebpb; Junb; Nfkbia; Ptgs2; Socs3
	rno04350	TGF-beta signaling pathway	Dcn; E2f5; Fst; Rbl1; Thbs1
	rno04064	NF-kappa B signaling pathway	Bcl2a1; Cd14; Lbp; Nfkbia; Ptgs2
	rno04620	Toll-like receptor signaling pathway	Cd14; Lbp; Nfkbia; Spp1; Tlr8
	rno04068	FoxO signaling pathway	Ccnb1; Ccnb2; Klf2; Plk1; Plk4; Sgk1
	rno04210	Apoptosis	Bcl2a1; Birc5; Ctsc; Lmnb1; Nfkbia; Ngf
	rno04658	Th1 and Th2 cell differentiation	Cd4; Lat; Mapk11; Rbpjl
	rno04620	Toll-like receptor signaling pathway	Ccl5; Irf3; Mapk11; Tlr9
	rno04514	Cell adhesion molecules (CAMs)	Cd4; RT1-A1; RT1-A2; RT1-CE10; RT1-CE4; RT1-M6-1
	rno04621	NOD-like receptor signaling pathway	Ccl5; Irf3; Mapk11; Oas1b; Trpm2
7 Day	rno04514	Cell adhesion molecules (CAMs)	Cd4; Cd40; Cd6; Cd8a; Cd8b; RT1-DMa; Sdc1; Selp
	rno04612	Antigen processing and presentation	Cd4; Cd8a; Cd8b; RT1-DMa
	rno04660	T cell receptor signaling pathway	Cd3d; Cd4; Cd8a; Cd8b
	rno04658	Th1 and Th2 cell differentiation	Cd3d; Cd4; RT1-DMa
	rno04064	NF-kappa B signaling pathway	Bcl2a1; Cd14; Cd40
	rno04659	Th17 cell differentiation	Cd3d; Cd4; RT1-DMa
14 Day	rno04514	Cell adhesion molecules (CAMs)	PVR; RT1-DMa; RT1-DMb
28 Day	rno04080	Neuroactive ligand-receptor interaction	Chrm2; Galr1; Glra1; Grin2a; Npffr1
	rno04151	PI3K-Akt signaling pathway	Chrm2; Itga1; Itga11; Tnr
